# Supplementary material for: CXCL2 combined with HVJ-E suppresses tumor growth and lung metastasis in breast cancer and enhances anti-PD-1 antibody therapy
Source: Mol Ther Oncolytics. 2020 Dec 25;20:175–86. doi: 10.1016/j.omto.2020.12.011 (PMC7851488; doi:10.1016/j.omto.2020.12.011)
Supplement: Document S1. Figures S1–S7 and Supplemental Materials and Methods [file mmc1.pdf]

OMTO, Volume *20*

Supplemental Information

CXCL2 combined with HVJ-E suppresses tumor growth and lung metastasis in breast cancer and enhances anti-PD-1 antibody therapy

Yi Chun Pan, Tomoyuki Nishikawa, Chin Yang Chang, Jiayu A. Tai, and Yasufumi Kaneda

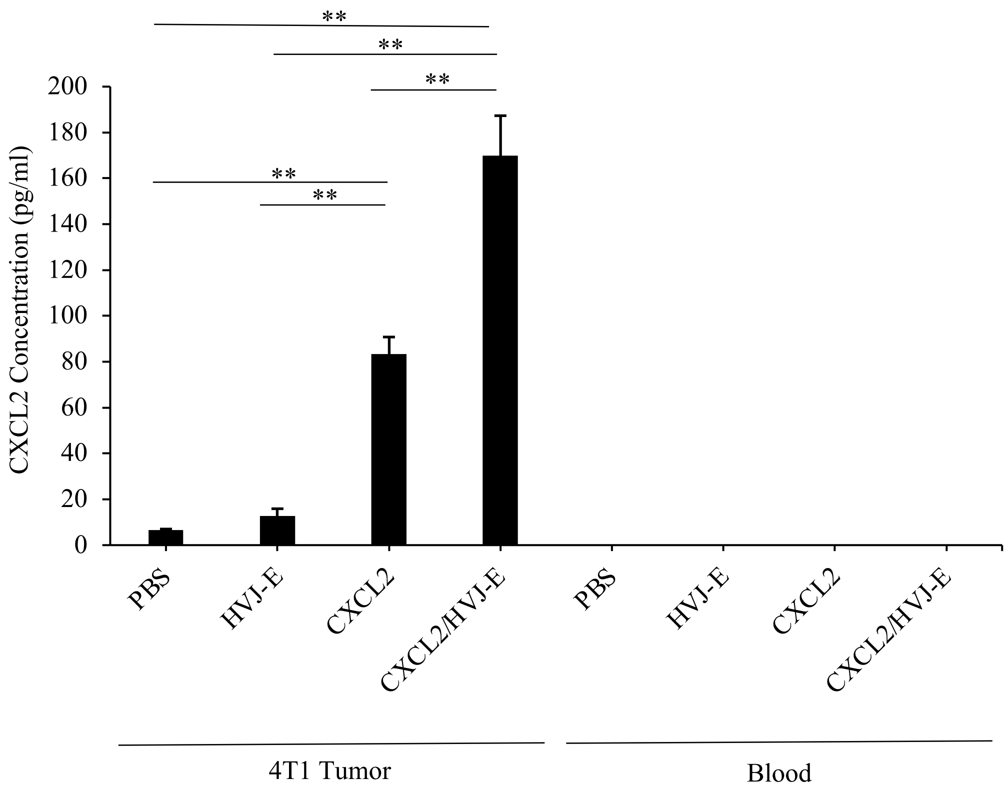

Figure. S1 CXCL2 in combination with HVJ-E increased CXCL2 in 4T1 tumor

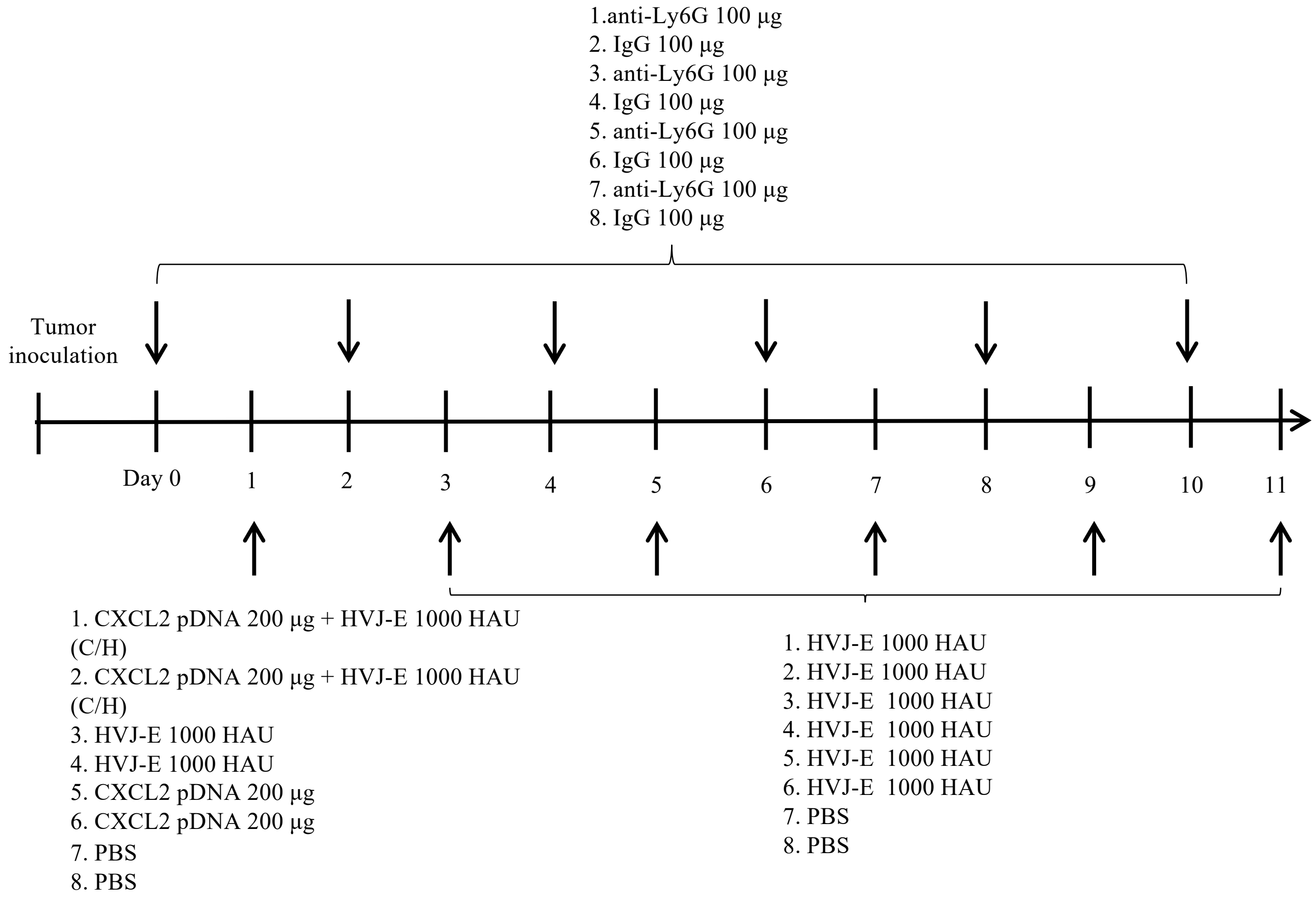

Figure. S2 The protocol for each treatment

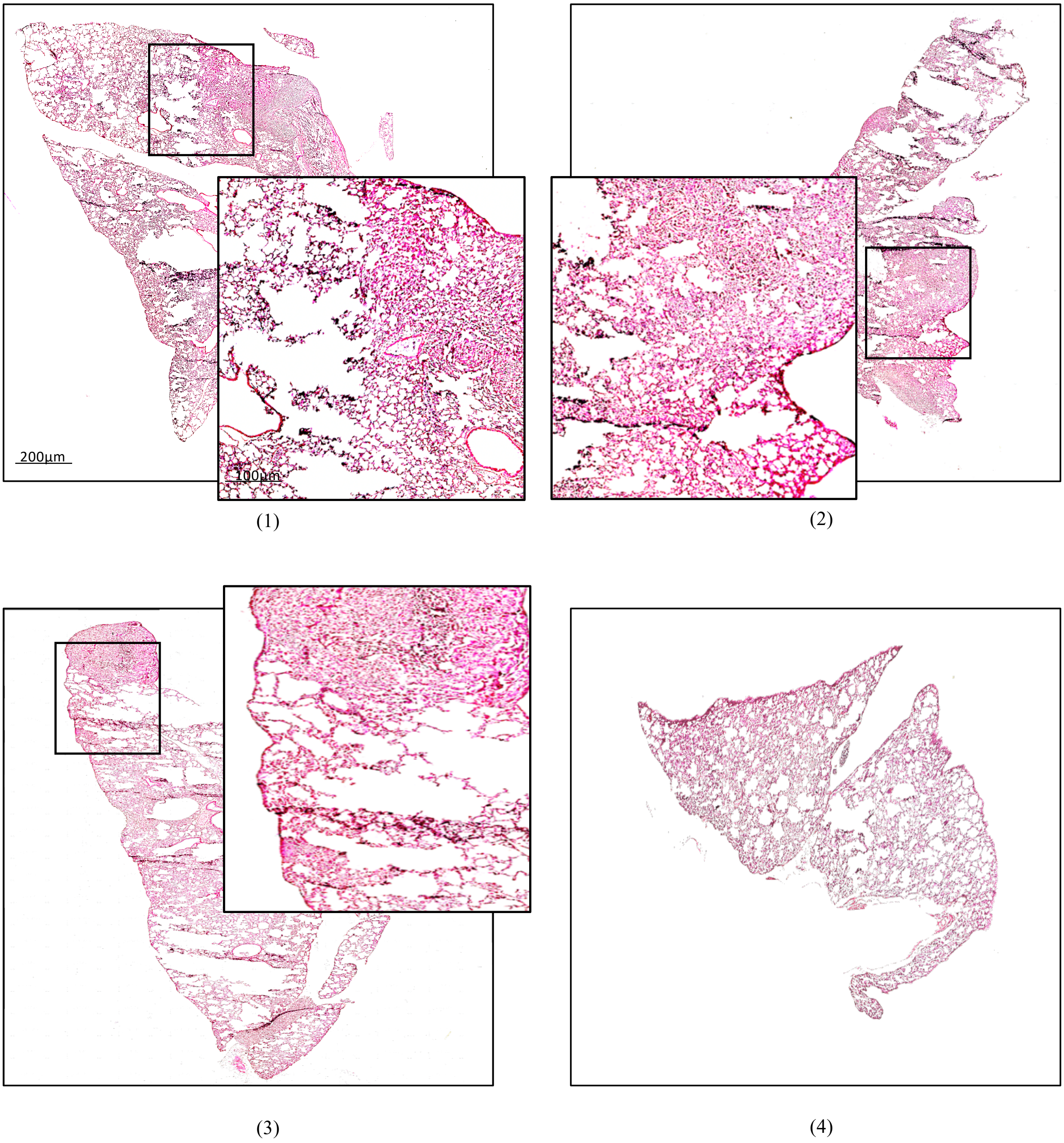

Figure.S3 C/H treatment suppressed 4T1 tumor metastasis to mouse lung

A

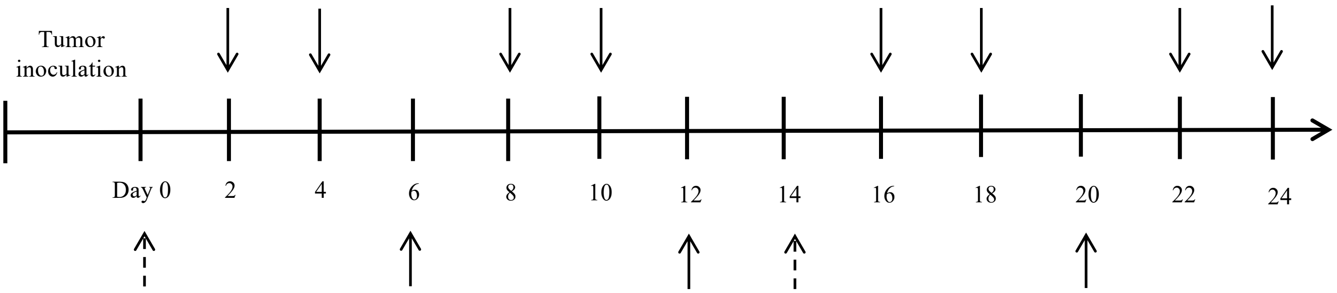

1. CXCL2 pDNA 200 μg + HVJ-E 1000 HAU (C/H) + anti-PD-1 Ab (250 μg)
2. CXCL2 pDNA 200 μg + HVJ-E 1000 HAU (C/H) + Ig G (250 μg)
3. CXCL2 pDNA 200 μg + HVJ-E 1000 HAU (C/H) + PBS
4. PBS + anti-PD-1 Ab (250 μg)
5. PBS + Ig G (250 μg)

1. anti-PD-1 Ab (250 μg)
2. Ig G (250 μg)
3. PBS
4. anti-PD-1 Ab (250 μg)
5. Ig G (250 μg)

1. HVJ-E 1000 HAU
2. HVJ-E 1000 HAU
3. HVJ-E 1000 HAU
4. PBS
5. PBS

B

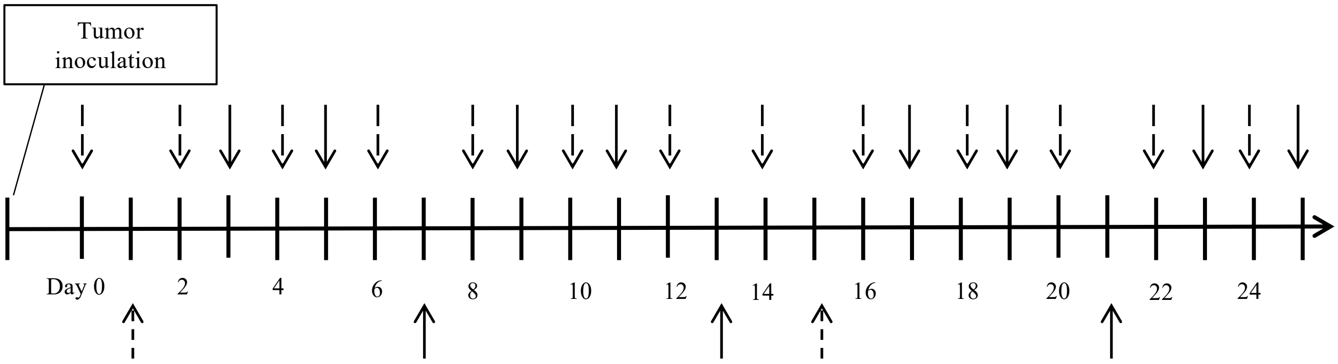

1. CXCL2 pDNA 200 μg + HVJ-E 1000 HAU (C/H) + anti-PD-1 Ab (250 μg)
2. CXCL2 pDNA 200 μg + HVJ-E 1000 HAU (C/H) + anti-PD-1 Ab (250 μg)
3. CXCL2 pDNA 200 μg + HVJ-E 1000 HAU (C/H) + anti-PD-1 Ab (250 μg)

1. anti-PD-1 Ab (250 μg)
2. anti-PD-1 Ab (250 μg)
3. anti-PD-1 Ab (250 μg)

1. anti-Ly6G 100 μg
2. IgG 100 μg
3. PBS

1. HVJ-E 1000 HAU
2. HVJ-E 1000 HAU
3. HVJ-E 1000 HAU

Figure. S4 The schedule of tumor treatment

A 4T1 Tumor

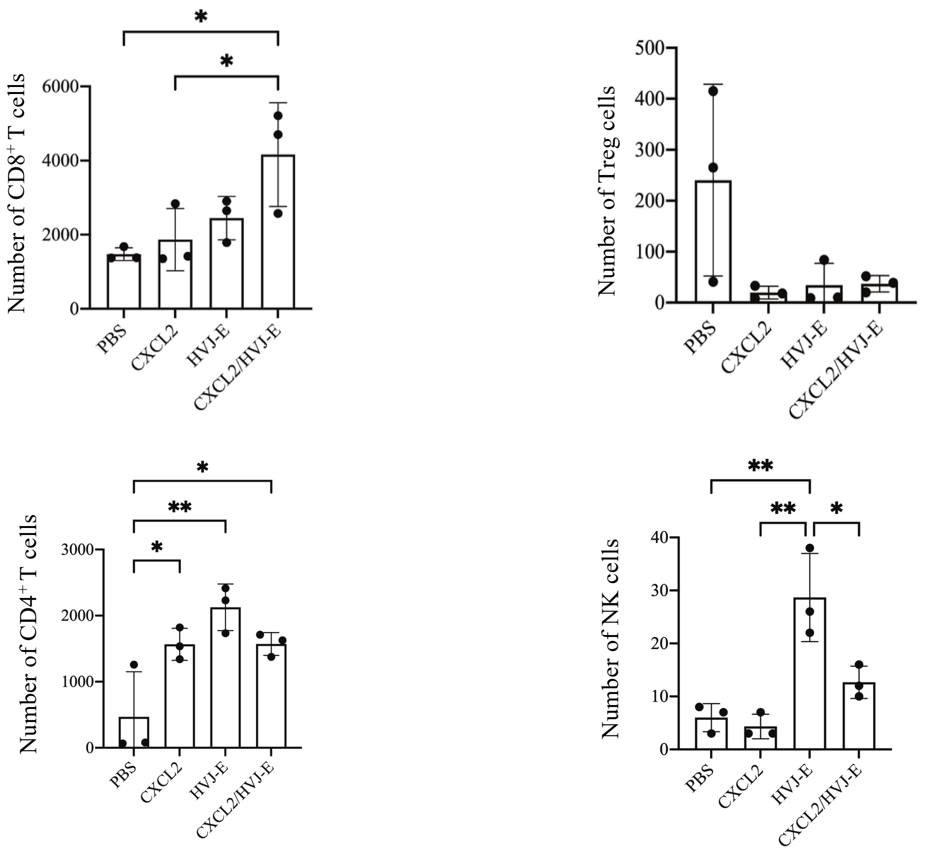

B 4T1 Lung

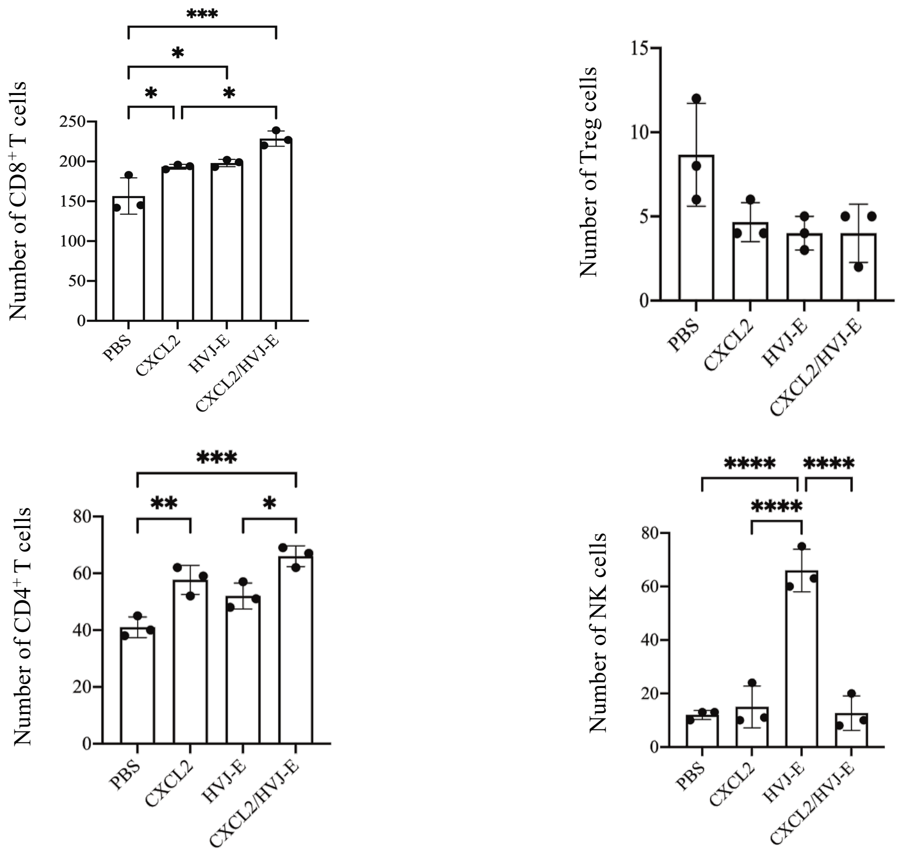

Figure. S5 C/H treatment induced CD8+ T cells in tumor and lung.

4T1 Tumor

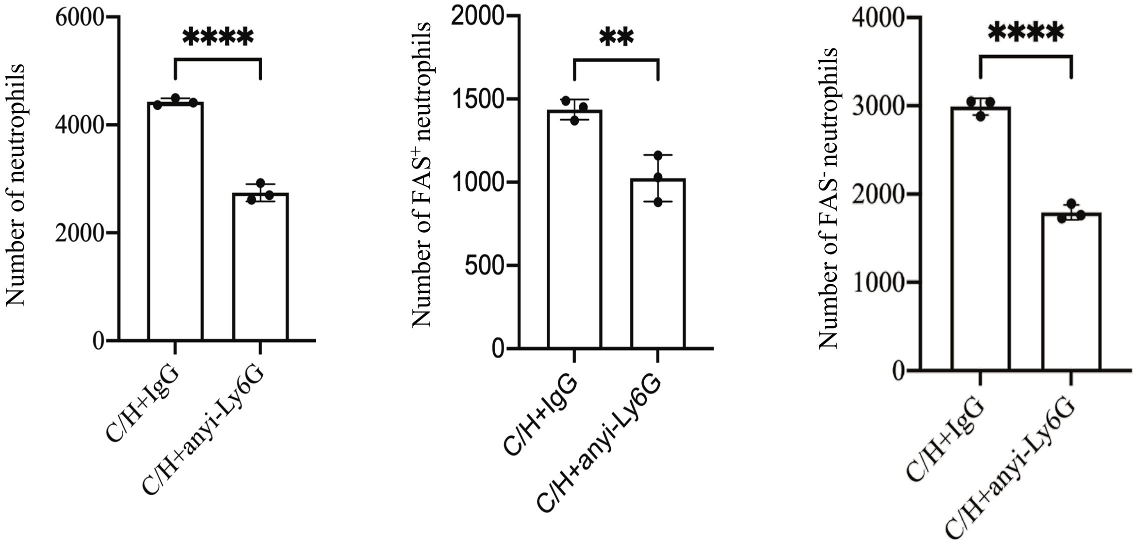

4T1 Lung

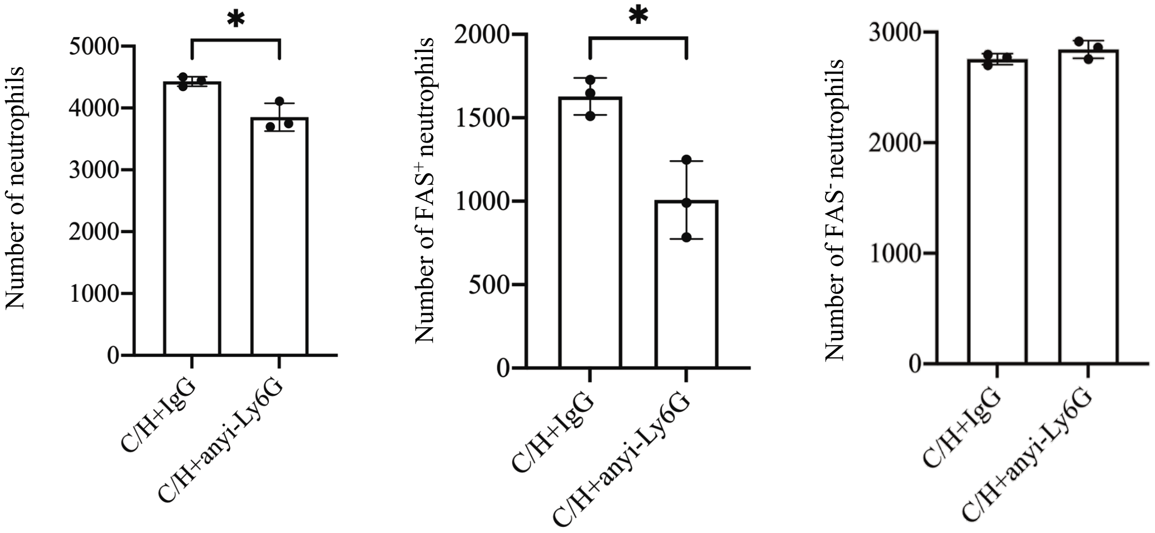

Figure. S6 The influence on C/H treatment with anti-Ly6G in 4T1 tumor and lung.

A

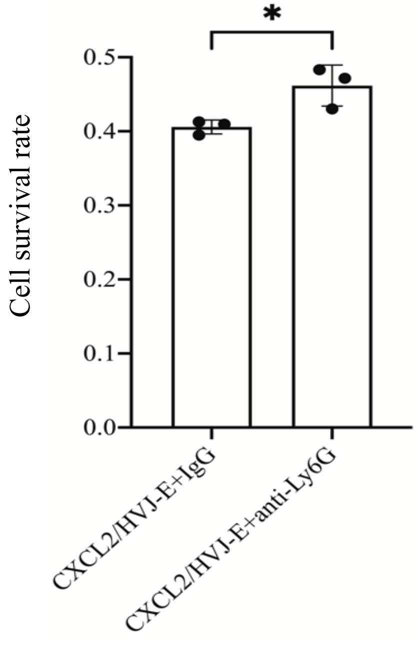

B

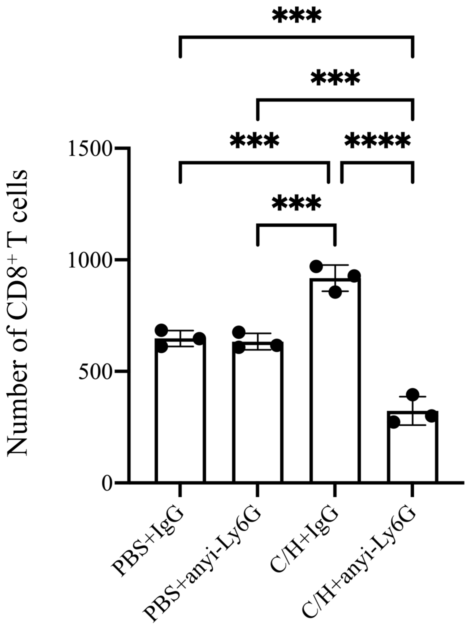

Figure. S7 **C/H treatment increased CD8^{+} T cells and induced CTLs activation.**

Figure. S1

CXCL2 in combination with HVJ-E increased CXCL2 in 4T1 tumor. The expression of CXCL2 in 4T1 tumors treated with PBS, HVJ-E, CXCL2 plasmid DNA, or C/H was measured with a CXCL2 ELISA. The CXCL2 protein level in the blood of 4T1 tumor-bearing mice was also measured. Mouse model measured by an ELISA assay. The data are shown as the mean ± SD (n = 4 per group). ** and * indicate $p < 0.01$ and $p < 0.05$, respectively.

Figure. S2

The protocol for each treatment. The protocol for each treatment is shown. The schedule of anti-Ly6G antibody administration in the 4T1 tumor mouse model is also shown.

Figure. S3

C/H treatment suppressed 4T1 tumor metastasis in the mouse lung. After each treatment, 4T1 tumor-bearing mice were sacrificed, and the lungs were analyzed by HE staining. (1), (2), (3), and (4) indicate the PBS treatment, CXCL2 plasmid DNA treatment, HVJ-E treatment, and C/H treatment, respectively. Lung tissues with metastasizing foci were magnified in (1), (2) and (3).

Figure. S4

The tumor treatment schedule. (A) The schedule of C/H treatment combined with anti-PD-1 antibodies in a 4T1 tumor mouse model is described. (B) The schedule of anti-Ly6G antibody administration on C/H treatment combined with anti-PD-1 antibodies in a 4T1 tumor mouse model is described.

Figure. S5

The number of immune cells in 4T1 primary and lung metastatic lesions. (A) The number of CD8+ T cells, CD4+ T cells, Treg cells and NK cells in tumors 24 hours after the final treatment were measured by flow cytometry. (B) The number of CD8+ T cells, CD4+ T cells, Treg cells ($CD4^+$, $CD25^+$) and NK cells ($CD49b^+$, $NKp46^+$) in lungs 1 week after the final treatment were measured by flow cytometry. The mean ± SD (n = 3 per group) was shown. ∗, ∗∗, ∗∗∗ and ∗∗∗∗ indicate $p < 0.05$, $p < 0.01$, $p < 0.001$ and $p < 0.0001$, respectively.

Figure. S6

The influence of anti-Ly6G antibody on neutrophil infiltration in 4T1 primary and lung metastatic lesions treated with C/H. The number of neutrophils ($CD11b^+$, $Ly\text{-}6G^+$), Fas^+ neutrophils ($CD11b^+$, $Ly\text{-}6G^+$, Fas^+) and Fas^- neutrophils ($CD11b^+$, $Ly\text{-}6G^+$, Fas^-) in 4T1 tumors (upper figures) and lungs (under figures) 24 hours or 1 week after the C/H treatment with anti-Ly6G or control IgG were analyzed by flow cytometry. ∗, ∗∗ and ∗∗∗∗ indicate $p < 0.05$, $p < 0.01$and $p < 0.0001$, respectively.

Figure. S7

C/H treatment increased $CD8^+$ T cells and induced CTLs activation. (A) The effect of anti-Ly6G antibody administration on $CD8^+$ T cell-mediated cell killing was compared with that of control IgG administration in 4T1 tumor model treated with C/H. (B) The number of CD8+ T cells in tumors 24 hours after PBS treatment and C/H treatment using anti-Ly6G or control IgG were measured by flow cytometry. The mean ± SD (n = 3 per group). ∗ and ∗∗∗ indicate $p < 0.05$ and $p < 0.001$, respectively.

Supplemental Materials and Methods

Analysis of CXCL2 protein expression of tumor tissues and blood by CXCL2 ELISA assay

Tumor tissues and blood were collected from 4T1 tumor-bearing mice 48 hours after the final treatment. The collected tissues were submerged in RIPA buffer (Nacalai Tesque Inc., Kyoto, Japan) at 2500 rpm for 20 seconds using a Multi-Beads Shocker (Yasui Kikai Co. Osaka, Japan). After homogenization on ice for 1 hour, the lysate was centrifuged at 15,000 x rpm for 10 minutes. The samples were frozen at -80° C. The collected blood was maintained at room temperature for 1 hour and centrifuged at 12 x G for 10 minutes. The serum was saved and frozen at -80° C. The samples were thawed and centrifuged at 10,000 x G for 5 minutes to remove cell debris. Tissue lysates containing equal amounts of protein (400 μg) and serum (25 μl) were subsequently used. The CXCL2 ELISA assay used a Mouse CXCL2 ELISA kit (MM200, R&D Systems, Minneapolis, USA) depending on the maker's protocol. A 96-well Mithras LB 940 Multimode Microplate Reader (Berthold Technologies GmbH & Co. KG, Bad Wildbad, Germany) was used to measure the result at 540 nm.

H&E staining

After the 4T1-bearing mouse model was treated with the C/H, HVJ-E, CXCL2 pDNA, or PBS treatments for three weeks, lung sections were fixed with 4% paraformaldehyde solution, embedded in OCT compound (Sakura Finetek Japan Co., Ltd., Tokyo, Japan), and sectioned at -80° C. Histological examinations were performed based on HE staining. HE staining procedures were subsequently performed on dehydrated sections and observed using a microscope (BZ-X700; Keyence, Osaka, Japan).

Supplemental Materials and Methods

Flow cytometry analysis of the tumors and lungs

Tumors were collected from the mice and minced into fine pieces in a digestion buffer containing 2% FBS and 2.5 mg/ml collagenase A (Roche, Basel, Switzerland). The samples were incubated in the digestion buffer at 37° C for 1 hour with a shaker, filtered through a 70-μm filter, and washed twice with PBS. The lungs were collected from the mice and minced into fine pieces in a digestion buffer containing 2% FBS and 1.5 mg/ml collagenase B (Roche, Basel, Switzerland). The samples were incubated in the digestion buffer at 37° C for 45 min with a shaker, filtered through a 70-μm filter, hemolyzed in hemolysis buffer (Immuno-Biological Laboratories Co., Ltd.), and washed twice with PBS. The collected cells were stained with the following fluorescent labeled antibodies: CD45 (Clone: 30-F11, 103134, Biolegend, Inc., San Diego, CA), CD3 (Clone: 17A2, 100236, Biolegend), CD8 (Clone: 53-6.7, 100726, Biolegend), CD4 (Clone: RM4-5, 100509, Biolegend), CD25 (Clone: 3C7, 101915, Biolegend), CD49b (Clone: DX5, 108907, Biolegend), NKp46 (Clone: 29A1.4, 137607, Biolegend), CD11b (Clone: M1/70, 101216, Biolegend), Ly6G (Clone: 1A8, 127614, Biolegend), and FAS (Clone: Jo2, eBioscience, San Diego, USA). All flow cytometry was performed on a Cyto FLEX S System B2-R3-V4-Y4 (BECKMAN COULTER, USA), and the analyses were performed using FlowJo software (FlowJo LLC, OR, USA).
