# Supplementary material for: CXCL2 combined with HVJ-E suppresses tumor growth and lung metastasis in breast cancer and enhances anti-PD-1 antibody therapy
Source: Mol Ther Oncolytics. 2020 Dec 25;20:175–86. doi: 10.1016/j.omto.2020.12.011 (PMC7851488; doi:10.1016/j.omto.2020.12.011)
Supplement: Document S2. Article plus supplemental information [file mmc2.pdf]

Molecular Therapy
Oncolytics
Original Article

# CXCL2 combined with HVJ-E suppresses tumor growth and lung metastasis in breast cancer and enhances anti-PD-1 antibody therapy

Yi Chun Pan,[1] Tomoyuki Nishikawa,[2] Chin Yang Chang,[2] Jiayu A. Tai,[2] and Yasufumi Kaneda[3]

[1]Division of Gene Therapy Science, Graduate School of Medicine, Osaka University, Osaka 565-0871, Japan; [2]Department of Device Application for Molecular Therapeutics, Graduate School of Medicine, Osaka University, Osaka 565-0871, Japan; [3]Head Quarter, Osaka University, Osaka 565-0871, Japan

**Breast cancer has a high risk of metastasis; however, no effective treatment has been established. We developed a novel immunotherapy for breast cancer to enhance cytotoxic T lymphocytes against cancer cells using N1-type neutrophils with anti-tumor properties. For this purpose, we combined CXCL2 (CXC chemokine ligand 2) plasmid DNA with inactivated Sendai virus (hemagglutinating virus of Japan)-envelope (HVJ-E). The combination of CXCL2 DNA and HVJ-E (C/H) suppressed the growth of murine breast cancers in orthotopic syngeneic models by enhancing cytotoxic T lymphocytes and inhibited lung metastasis of breast cancer from primary lesions. N1-type neutrophils (CD11b$^+$ Ly6G$^+$ FAS$^+$) increased in the tumor microenvironment with C/H treatment, and tumor suppression and cytotoxic T lymphocyte activation from C/H was blocked after administrating anti-neutrophil antibodies, which indicates the role of N1-type neutrophils in cancer immunotherapy. We also demonstrated that the anti-tumor activities of C/H treatment were enhanced by the administration of anti-PD-1 antibodies through neutrophil-mediated cytotoxic T lymphocyte activation. Thus, the triple combination of C/H and anti-PD-1 antibody C/H treatment may provide an improvement in cancer immunotherapy.**

## INTRODUCTION

With the advancements of medical technology, the cure rate of breast cancer is continually increasing. However, although the 5-year survival rate of breast cancer was as high as 99% for local cancer from 2001 to 2007 in the United States, this rate drops to 23% if distant metastasis occurs.[1] Thus, breast cancer remains the second most deadly cancer in women and determining a treatment for metastatic breast cancer is a significant challenge.

Recently, immune therapy has become the fourth most common cancer treatment in addition to surgery, chemotherapy, and radiotherapy. Immune checkpoints such as PD-1/PD-L1 and CTLA-4 play important roles in cancer immune therapy. In an immunosuppressive microenvironment, tumors or immune cells can overexpress checkpoints, resulting in immune tolerance and escape.[2] Therefore, blocking immune checkpoints is a new immunotherapy for cancer. Lately, some studies have reported that anti-PD-1/PD-L1 is effective against melanoma,[3–5] non-small cell lung carcinoma (NSCLC),[4,5] renal cancer,[5,6] Hodgkin lymphoma,[5,7,8] etc. However, the efficacy of immune checkpoint inhibitory therapy is not as high as expected in several types of cancers. For example, approximately 70% of melanoma patients who received anti-PD-1 antibodies displayed stable disease (SD) or progressive disease (PD).[9] Thus, cancer immunotherapy is currently focused on how to prevent resistance to immune checkpoint antibody therapy. In some solid cancers, such as breast cancer, which is not highly sensitive to anti-PD-1 antibodies, Bertucci et al.[10] suggest using anti-PD-1/PD-L1 in combination with other checkpoint inhibitors or chemotherapy, targeted therapy, radiotherapy, or with novel immunotherapies to increase the efficacy of immunotherapies in breast cancers.

We have reported multiple anti-tumor activities of inactivated Sendai virus (hemagglutinating virus of Japan; HVJ)-envelope (HVJ-E), such as the activation of anti-tumor immunity and the induction of cancer-specific cell death.[11–14] Various combinations of cancer treatments with HVJ-E have been tested to enhance its anti-tumor activities.[15,16] Among them, the combination of poly I:C with HVJ-E synergistically increased anti-tumor immunity, and CXCL2 upregulation by poly I:C was a key molecule for enhancing the anti-tumor immunity of HVJ-E.[16]

CXCL2 (CXC chemokine ligand 2) is produced by mast cells and macrophages and can recruit neutrophils.[17,18] Some studies have shown that neutrophils play anti-tumor or pro-tumor roles in the tumor microenvironment (TME).[19–21] Similar to tumor-associated macrophages, which have a classic (M1) and alternative (M2) form, tumor-associated neutrophils (TANs) also have anti-tumorigenic N1 neutrophils and pro-tumorigenic N2 neutrophils.[22–25] Recent research has noted that the N2 phenotype can cause transforming growth factor (TGF)-β to block tumor inhibition and decrease CD8$^+$ T cell activation.[26] TANs are associated with tumor progression

Received 31 July 2020; accepted 22 December 2020;
https://doi.org/10.1016/j.omto.2020.12.011.

**Correspondence:** Yasufumi Kaneda, Head Quarter, Osaka University, Osaka 565-0871, Japan.
**E-mail:** kaneday@gts.med.osaka-u.ac.jp


This is an open access article under the CC BY license (http://creativecommons.org/licenses/by/4.0/).

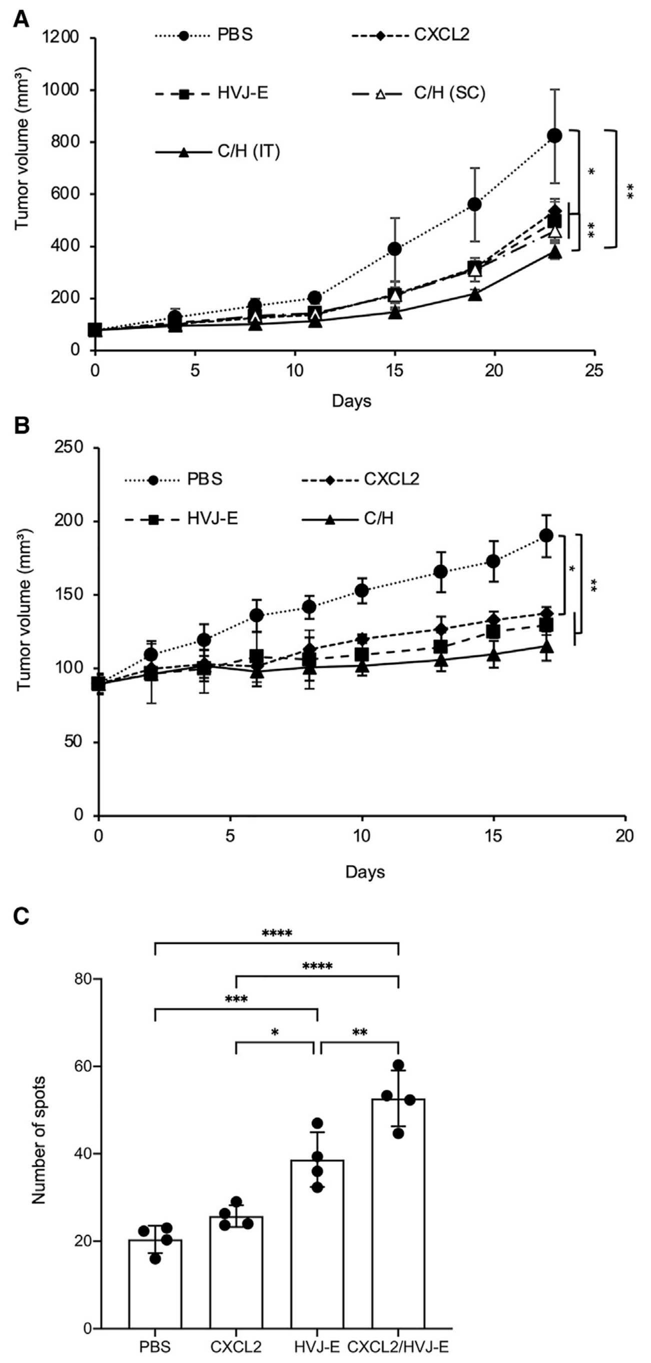


**Figure 1. CXCL2 in combination with HVJ-E treatment suppressed tumor growth and induced a 4T1 tumor-specific INF-γ response**

(A and B) 4T1 (A) or BALB-MC.E12 (B) cells were intradermally implanted on the back of BALB/c mice. Those mice were treated intratumorally (IT) with CXCL2 plasmid DNA (pCXCL2), HVJ-E, or pCXCL2 in combination with HVJ-E (C/H) at day 0, followed by five additional treatments of HVJ-E at days 2, 4, 6, 8, and 10. C/H (s.c.) indicates a subcutaneous injection of C/H following HVJ-E. The specific comparison was C/H (s.c.), pCXCL2, and HVJ-E treatment with C/H treatment. The means ± SD of tumor volumes calculated from the diameter of the tumor mass are presented (n = 4 per group). (C) Tumor-specific INF-γ-secreting T cells were measured with an ELISpot assay. Values are stated as the mean ± SD (n = 4 per group). $^{*}p < 0.05$, $^{**}p < 0.01$, $^{***}p < 0.001$, and $^{****}p < 0.0001$.

of angiogenesis and metastasis.[25,27] Although Eruslanov et al.[28] showed that TANs can stimulate T cell responses in the early stages of lung cancer, another study reported that neutrophils can inhibit tumor growth and delay metastases by directly suppressing or regulating the immune system.[29] Therefore, properly stimulated TANs are expected to inhibit tumor growth.

We discovered that HVJ-E directly and indirectly increased the N1 neutrophil population, which enhanced cytotoxic T lymphocyte (CTL) activation against cancers in the TME.[16] We then developed a new gene therapy against cancers by combining CXCL2 plasmid DNA and HVJ-E (C/H).

Here, we examined the anti-tumor activities of C/H in murine breast cancer syngeneic models, including an orthotopic model; we also examined the inhibition of spontaneous lung metastasis of breast cancer from a primary tumor mass. C/H enhanced the tumor suppression effect of anti-PD-1 antibody treatment in a breast cancer model. Our findings indicated that the triple combination of C/H and anti-PD-1 antibodies may be an improvement in cancer immunotherapy.

## RESULTS

### CXCL2 in combination with HVJ-E treatment suppressed tumor growth and induced a tumor-specific interferon (IFN)-γ response

To prove whether CXCL2 combined with HVJ-E treatment affects breast cancer tumor growth, we first used a 4T1 tumor-bearing mouse model that was treated intratumorally (IT) or subcutaneously (s.c.) with CXCL2 plasmid DNA (pCXCL2), HVJ-E, or pCXCL2 in combination with HVJ-E once, followed by five additional treatments of HVJ-E every other day. Although we have reported that HVJ-E can incorporate plasmid DNA via treatment with low concentrations of Triton X-100,[30] in the current protocol, C/H is the mixture of pCXCL2 and HVJ-E that is not being incorporated in clinical trials.

C/H was more effective for tumor reduction than either CXCL2 or HVJ-E alone (Figure 1A). However, the subcutaneous injection of C/H was not as effective at tumor suppression as the IT injection (Figure 1A). In the treatment of other cancer models using the same therapeutic protocol, C/H was the most effective in a mouse xenograft model of BALB-M.EC12 murine breast cancer cells (Figure 1B). When CXCL2 protein expression was examined in the tumor mass, CXCL2 was detected in tumors by both pCXCL2 and C/H, with C/H resulting in expression levels more than twice as high. No CXCL2 was detected in the blood (Figure S1).

Previous studies have suggested that HVJ-E plays an important role in T cell activation,[13,14] and pCXCL2-incorporated HVJ-E suppressed B16-F10 melanoma.[16] Our result showed C/H suppressed 4T1 tumor

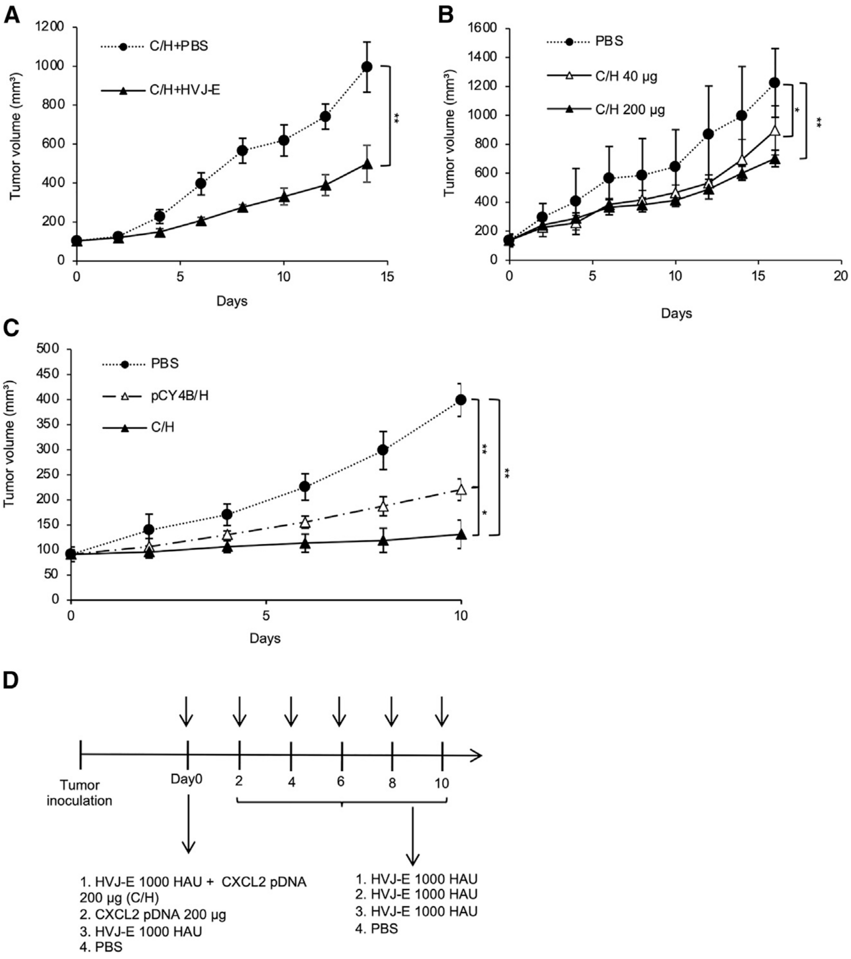


**Figure 2. The optimum conditions of C/H treatment for tumor suppression**

(A) 4T1 tumor-bearing mice were treated with C/H once, followed by HVJ-E or PBS five times (six total injections) every other day. (B) 4T1 tumor-bearing mice were treated IT with PBS or pCXCL2 (40 or 200 μg) in combination with HVJ-E at day 0, followed by five additional treatments of HVJ-E at days 2, 4, 6, 8, and 10. (C) 4T1 tumor-bearing mice were IT treated with PBS, C/H, or pCY4B/H. (D) Tumor treatment protocol. 4T1 cells were implanted on the back of BALB/c mice. The mice were treated IT with PBS, pCXCL2 (200 μg), HVJ-E (1,000 HAU), or pCXCL2 (200 μg) in combination with HVJ-E (C/H) at day 0, followed by five additional treatments of HVJ-E at days 2, 4, 6, 8, and 10. Tumor volumes were measured every 2–3 days. Tumor volumes are presented as the mean ± SD (n = 4 per group). *p < 0.05 and **p < 0.01.

growth, so we speculated that C/H treatment might have involved T cell activation. The IFN-γ enzyme-linked immune absorbent spot (ELISpot) assay revealed that mice treated with C/H had significantly increased IFN-γ-producing splenocytes compared with other treatments (Figure 1C).

Together, these results show that C/H treatment inhibited 4T1 tumor growth and activated tumor-specific IFN-γ-secreting T cells.

### The optimum conditions of C/H treatment for tumor suppression

First, to determine whether five HVJ-E injections were necessary after C/H treatment (for a total of six HVJ-E injections), the effect of HVJ-E injections after C/H treatment was evaluated and compared with PBS injections. As shown in Figure 2A, tumor growth was significantly suppressed following HVJ-E injection. We did not alter the procedure involving five HVJ-E injections after C/H because our clinical trials had already determined the efficacy of six total HVJ-E injections. The amount of HVJ-E used in the mice was also determined based on the protocol of our melanoma clinical trials using HVJ-E alone.

Next, to determine the optional dosage of pCXCL2 in combination with HVJ-E (1,000 hemagglutinating unit [HAU]), we compared 40 or 200 μg of pCXCL2 combined with HVJ-E. Although 200 μg of pCXCL2 appeared to be more effective than 40 μg, there was no statistically significant difference in the suppression of tumor growth between 200 and 40 μg of pCXCL2 (Figure 2B).

Then, to examine the effect of CXCL2 cDNA on tumor suppression when combined with HVJ-E, we compared C/H with the combination of empty plasmid vector, pCY4B, and HVJ-E (pCY4B/H). C/H was more effective for tumor suppression than pCY4B/H without CXCL2 cDNA. Compared with HVJ-E combined with the control plasmid without CXCL2 cDNA, C/H significantly reduced tumor volume, indicating the need for CXCL2 cDNA (Figure 2C). Based on these results, the treatment protocol (C/H protocol) was established as shown in Figure 2D using a mixture of pCXCL2 (200 μg) and HVJ-E (1,000 HAU) once followed by five additional HVJ-E (1,000 HAU) treatments every other day for a total of six treatments.

### CXCL2 in combination with HVJ-E treatment induces N1 TANs, suppresses 4T1 tumor growth, and elevates CTL activation

CXCL2 functions mainly in the recruitment of neutrophils from the blood and in the activation and promotion of neutrophil function.[31] In tumors, neutrophils can be polarized into anti-tumorigenic N1 or more pro-tumorigenic N2 subtypes.[22,23] Our previous study showed that HVJ-E can polarize neutrophils to N1 neutrophils.[16] To investigate whether C/H can polarize neutrophils in 4T1 tumor-bearing mice, the N1 TAN population was analyzed using flow cytometry 24 h after the treatments. Our results showed that both the number of N1 TANs and the ratio of N1 TANs to total TANs increased significantly after treatment with the C/H protocol compared with the

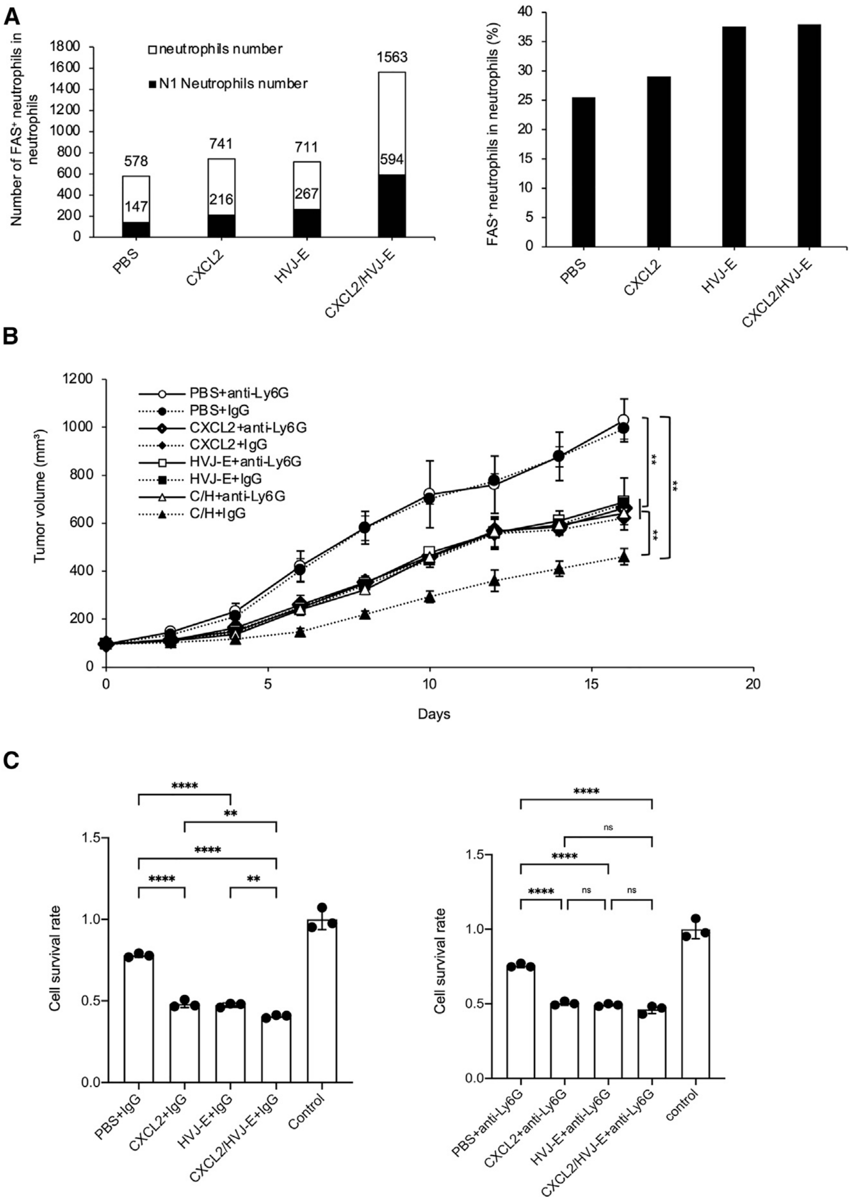


**Figure 3. C/H treatment induced N1 TANs suppressing 4T1 tumor growth and elevated CTL activation**

(A) The number of N1 neutrophils (CD11b$^+$, Ly-6G$^+$, Fas$^+$) and total neutrophils (CD11b$^+$, Ly-6G$^+$) in tumors 24 h after the final treatment was measured by flow cytometry. The left figure shows the number of N1 TANs and total TANs, and the right figure shows the ratio of N1 TANs to total TANs. (B) 4T1 tumor-bearing mice that were intraperitoneally injected with neutrophil-neutralizing antibodies (anti-Ly6G antibodies) or control IgG were IT treated with PBS, pCXCL2, HVJ-E, or C/H. We specifically compared C/H treatment using anti-Ly6G antibodies to using control IgG. The tumor volumes are shown as the mean ± SD (n = 3 per group). (C) Mice from (B) were sacrificed 1 week after the last treatment, and CD8$^+$ T cells were isolated from spleens that were co-cultured with 4T1 cells at a 50:1 ratio for 24 h. Then, cell survival was measured by an MTS assay. The mean ± SD (n = 3 per group) was shown. **p < 0.01 and ****p < 0.0001. NS, not significant; Control, 4T1 without treatment.

treatment using PBS, pCXCL2, or HVJ-E (Figure 3A; Figure S5A). Some studies have shown that N1 TANs can suppress tumor growth.[16,27,28] To confirm whether neutrophils were involved in the inhibition of tumor growth, a neutrophil-blocking experiment was performed using anti-Ly6G antibodies based on the protocol shown in Figure S2. The results indicated that anti-Ly6G administration significantly abolished the tumor suppression effect of the treatment with the C/H protocol using control immunoglobulin G (IgG) (Figure 3B).

Next, we examined whether the tumor-killing activity of CTLs from splenocytes was affected by anti-Ly6G administration. C/H treatment using control IgG killed significantly more tumor cells than the other treatments using control IgG. However, the killing activity of the C/H treatment was lost with the administration of anti-Ly6G antibodies (Figure 3C; Figure S7A). Moreover, we also examined the population of CD8$^+$ T cells in mouse tumor and found depleting Ly6G$^+$ cells can decrease the population of CD8$^+$ T cells (Figure S7B).

These results suggest that the tumor suppression and tumor-killing activities of CTLs during treatment with the C/H protocol were mediated by N1 TANs.

## C/H treatment suppressed lung metastasis in an orthotopic 4T1 tumor model

4T1 breast cancer tumor cells are highly tumorigenic and invasive, with a high risk of metastasis from the primary tumor to distant sites such as the lungs, brain, bone, liver, blood, and lymph nodes.[32,33] To prove whether C/H can affect 4T1 tumor metastasis in mouse lungs, 4T1-Luc cells were intradermally inoculated into the fourth mammary gland of BALB/c mice. The primary lesion was treated with pCXCL2, HVJ-E, or C/H once, followed by five additional treatments of HVJ-E every 2 days for a total of six treatments. Similar to Figure 1A, mice receiving the C/H treatment showed significant tumor growth suppression of the primary lesion compared with the other treatments (Figure 4A). One week after the last treatment, we analyzed luciferase activity in the lungs of the treated mice. Luciferase activity in the lungs of the mice treated with C/H was significantly reduced compared with that in mice receiving other treatments (Figure 4B). The hematoxylin

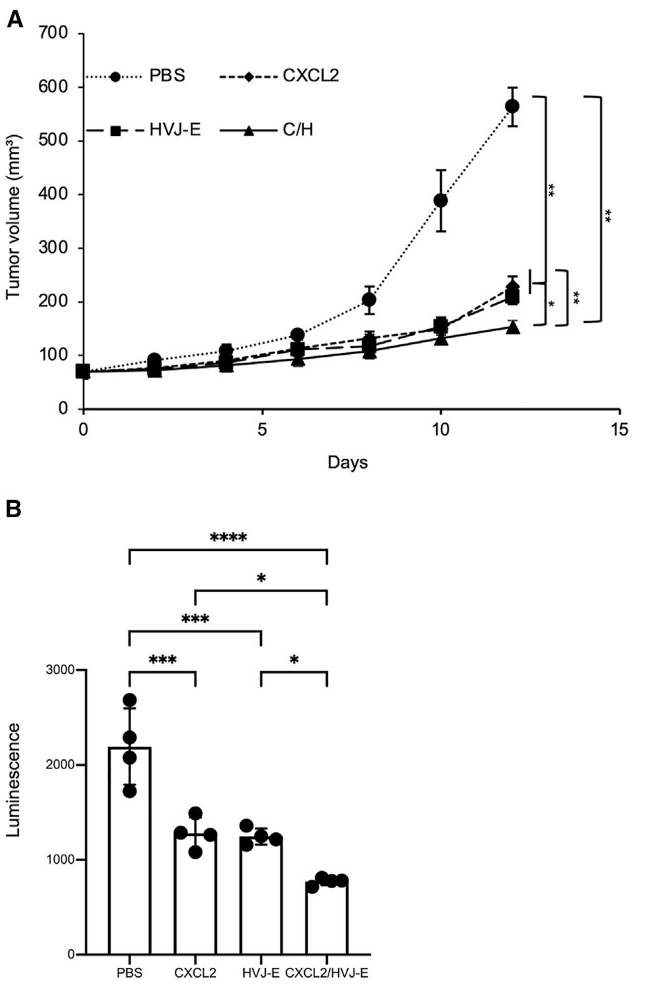


**Figure 4. C/H treatment suppressed lung metastasis in an orthotopic 4T1 tumor model**

(A) 4T1-luc bearing mice were treated with C/H, HVJ-E, CXCL2 plasmid DNA, or PBS at day 0, followed by five treatments with HVJ-E (1,000 HAU) or PBS at days 2, 4, 6, 8, and 10. Tumor volumes are shown as the mean ± SD (n = 4 per group). (B) Luciferase assay of lungs from mice shown in (A) 1 week after the treatments. The data are shown as the mean ± SD (n = 4 per group). *p < 0.05, **p < 0.01, ***p < 0.001, and ****p < 0.0001.

and eosin (H&E) staining also indicated that after receiving C/H treatment in the primary tumor, there were no visible metastatic foci in the lung tissue (Figure S3). These results suggest that CXCL2 in combination with HVJ-E treatment suppressed the progression of mouse lung metastasis from 4T1 primary lesions.

### CXCL2 in combination with HVJ-E treatment induced N1 neutrophil infiltration in a 4T1 metastasis lung model

Some recent studies reported that TANs can stimulate T cell responses in early-stage human lung cancer.[28] To determine the mechanism of C/H treatment-mediated lung metastasis suppression, we investigated whether neutrophil infiltration into the lungs of mice was affected by C/H treatment. Mouse lungs were harvested 1 week after the final treatment. We found that C/H treatment increased the population of neutrophils in the lungs more than the other treatments (Figure 5A). Additionally, the number of N1 TANs per 10,000 lung cells increased significantly with C/H treatment (247) compared with CXCL2 treatment (13), HVJ-E treatment (80), or PBS treatment (4) (Figure 5B). We also checked other immune cells in the lungs, and the result showed C/H treatment increased total all neutrophils (Figure 5B) and CD4+ T cells in the lungs, while regulatory T cells (Tregs) and natural killer (NK) cells were not increased (Figure S5B). $CD8^+$ T cells were specifically increased compared with the CXCL2 treatment group but not with the HVJ-E treatment group (Figure S5B). Thus, C/H treatment in the primary lesion also induced N1 neutrophil infiltration into mouse lungs in the 4T1 metastasis model. This suggests that C/H treatment protected the lungs from metastasized tumor growth by N1-type neutrophil surveillance.

### C/H treatment improved the tumor suppression effect of anti-PD-1 antibody effectiveness in an orthotopic breast cancer model

4T1 breast cancer cells are used to create the triple-negative breast cancer model and have a poor prognosis due to complex immunosuppressive mechanisms in the TME.[34] The clinical response rates to immune checkpoint inhibition such as anti-PD-1 treatment remain low.[2] Therefore, we attempted to explore whether C/H treatment increased the tumor suppression activity of anti-PD-1 antibody treatment (Figure S4A). 4T1 cancer cells were implanted into mouse mammary glands. The orthotopic model mice were treated with anti-PD-1 antibodies or control IgG along with C/H treatment or PBS treatment. As shown in Figure 6A, anti-PD-1 antibodies alone had no suppression effect on tumor growth compared with PBS. However, the combination of anti-PD-1 antibodies with C/H treatment showed significant tumor growth inhibition compared with anti-PD-1 and PBS or control IgG and C/H treatment (Figure 6A). PD-1 blocking antibodies inhibit the interaction of PD-1 with both PD-L1 and PD-L2, resulting in enhanced T cell cytotoxicity.[3] To investigate whether T cell cytotoxicity was enhanced, we isolated $CD8^+$ T cells from mouse spleens 24 h after the last treatment and co-cultured them with 4T1 cells *in vitro*. Tumor cell survival was examined using the CellTiter 96® $AQ_{ueous}$ non-radioactive cell proliferation (MTS) assay. Our results showed that the tumor cell survival rate was significantly reduced with the combined anti-PD-1 antibody and C/H treatment, indicating that the anti-PD-1 antibody and C/H treatment enhanced CTL activity against 4T1 tumor cells (Figure 6B).

Next, to prove whether the tumor suppression effect of the triple combination of anti-PD-1 antibodies and C/H treatment was mediated by neutrophils, neutrophil-neutralizing antibodies were administered during the combination treatment (Figure S4B). The results showed that anti-Ly6G antibodies eliminated neutrophils. The tumor suppression effect of the combination treatment was significantly abolished with anti-Ly6G antibody administration (Figure 6C). Therefore, our results suggest that the enhancement of tumor suppression activity of anti-PD-1 antibody therapy combined with C/H

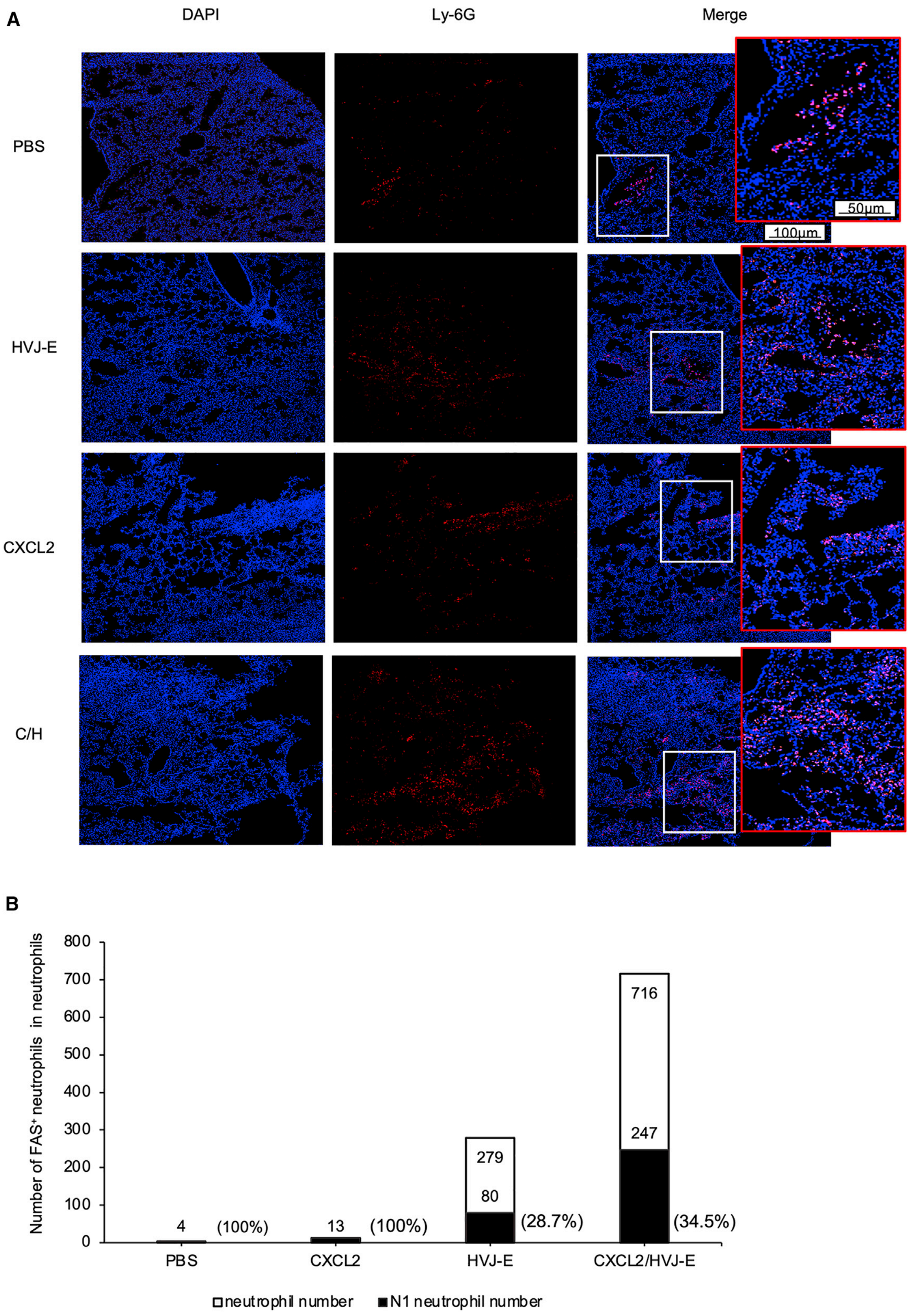


*(legend on next page)*

treatment was mediated by CTL activation, which was a result of the increase of N1 TANs.

## DISCUSSION

We report here that C/H inhibited breast cancer in mouse models and suppressed lung metastasis by increasing N1-type neutrophils in the TME. The C/H treatment enhanced the anti-tumor immunity of anti-PD-1 antibody therapy and anti-PD-1 antibody administration and increased the tumor suppression activity of C/H. The triple combination of C/H and anti-PD-1 antibodies provides a breakthrough in cancer immunotherapy, particularly for patients refractory to anti-PD-1 antibody therapy.

Immune checkpoint inhibitory therapy has been evaluated as an epoch-making cancer treatment. However, the analysis of many clinical cases has gradually indicated that more than half of patients are insensitive to this therapy, particularly patients with solid cancers.[10] The mechanism of this insensitivity has been investigated, and three conditions are thought to be necessary for anti-PD-1 antibody therapy to be effective: the infiltration of T cells into tumor tissue, the presence of immune cells expressing PD-1 and PD-L1 in tumor tissues, and the presence of a T cell population specifically recognizing tumor antigens.[5,35] To break the refractory condition, several combinations with immune checkpoint inhibitors have been evaluated, such as the combination of anti-PD-1 antibodies with anti-CTLA-4 or anti-Lag-3 antibodies.[5] Those combinations appeared to enhance tumor suppression compared with single antibody administration, but side effects were more frequent and in some cases more serious.[36,37] There may be limitations to accelerating CTL function by direct control of switches on T cells. Considering how cancers escape from immune surveillance, cancer cells modulate the TME to induce immune tolerance to themselves in the host immune system. To successfully provide immunotherapy to cancer patients, the TME must be remodeled to prevent immune tolerance.

In breast cancer in particular, some studies have shown that "inflamed" tumors, which are enriched with dendritic cells (DCs) and $CD8^+$ T cells, have an effective response to immunotherapy.[38] However, only a small percentage of breast cancers are considered "inflamed" tumors compared with other cancers,[38,39] because invasive breast cancer is rich in activated Tregs and has an effective inhibitory function.[39,40] Thus, breast cancers have low response to anti-PD-1 antibodies. This discovery suggests that reducing the activated Tregs or increasing the activation of DCs and $CD8^+$ T cells in the TME may be an efficient treatment.

We have developed an anti-tumor reagent using HVJ-E and discovered that HVJ-E itself has various anti-tumor activities, including the activation of anti-tumor immunity and the induction of cancer-cell-specific apoptosis.[13] To activate anti-tumor immunity, HVJ-E recruits T cells and NK cells to the TME by CXCL10 and activates those cells with IFN-β and -γ.[41] HVJ-E also inhibits Treg infiltration into the TME by IFN-β and suppresses Treg function with IL-6.[13] HVJ-E was originally developed as a gene therapy vector that can incorporate plasmid DNA into the vesicle via mild detergent treatment.[42] To enhance anti-tumor activity, gene therapy using the HVJ-E vector has been performed in various tumor models. Among them, we found that CXCL2 cDNA-incorporated HVJ-E enhanced anti-tumor immunity in a mouse melanoma model by increasing N1-type TANs.[16] In this manuscript, we utilized a mixture of HVJ-E and CXCL2 plasmid DNA without low concentrations of Triton X-100 incorporating plasmid DNA based on Pharmaceuticals and Medical Devices Agency (PMDA; Japanese Food and Drug Administration [FDA]) requirements. The mixture of HVJ-E and CXCL2 plasmid DNA succeeded in enhancing CXCL2 expression in the tumor mass and displayed anti-tumor activity by enhancing CTL activity against cancer cells, which resulted from the accumulation of N1-type TANs.

Some previous reports have described the function of TANs. Generally, naive neutrophils gradually turn into pro-tumorigenic N2-type TANs in the TME.[26] We have reported that HVJ-E polarizes both naive and N2-type neutrophils into N1-type neutrophils with anti-tumorigenic properties.[16] In our preliminary experiment, tumor killing activity was enhanced in $CD8^+$ T cells when mixed with HVJ-E-treated neutrophils, although the exact mechanism remains unknown. Thus, C/H treatment modulates the TME by recruiting neutrophils with CXCL2 and polarizes neutrophils into the N1 type via HVJ-E, which activates CTLs against cancer cells. Activated T cells upregulate the T cell-inhibitory signaling pathway and result in an exhausted state. The combination of C/H with anti-PD-1 antibodies activates CTLs via C/H and inhibits T cell exhaustion by blocking PD-1, which maintains killer T cell function. HVJ-E inhibits pro-tumorigenic properties in the TME by modulating both neutrophils[16] and macrophages (C.Y.C., unpublished data) in addition to Treg suppression. Moreover, HVJ-E induces the infiltration of T and NK cells into the TME, which enables CTLs to be easily accessible to cancer cells.[43]

As shown in Figure 4B, C/H treatment inhibited spontaneous lung metastasis from the primary tumor mass. Luciferase-expressing 4T1 tumor cells were used to quantitatively evaluate lung metastasis. To evaluate lung metastasis over time, IVIS imaging (in vivo imaging system) was conducted, but it was difficult to detect microscopic metastatic foci. There are two possibilities why luciferase expression indicating the presence of 4T1 tumor cells in the lungs was significantly

**Figure 5. C/H treatment induced N1 neutrophil infiltration in 4T1 metastatic lungs**

(A) Immunostaining of neutrophils (Ly-6G) of the mice lungs 1 week after the final treatment. The images in white frames represent a four-fold magnification of the images in red frames (n = 4 per group). (B) The number of N1 neutrophils ($CD11b^+$, $Ly\text{-}6G^+$, $Fas^+$) and total neutrophils ($CD11b^+$, $Ly\text{-}6G^+$) in the mouse lungs was measured by flow cytometry 1 week after the final treatment. The numbers in parentheses indicate the ratio of N1 neutrophils to total neutrophils.

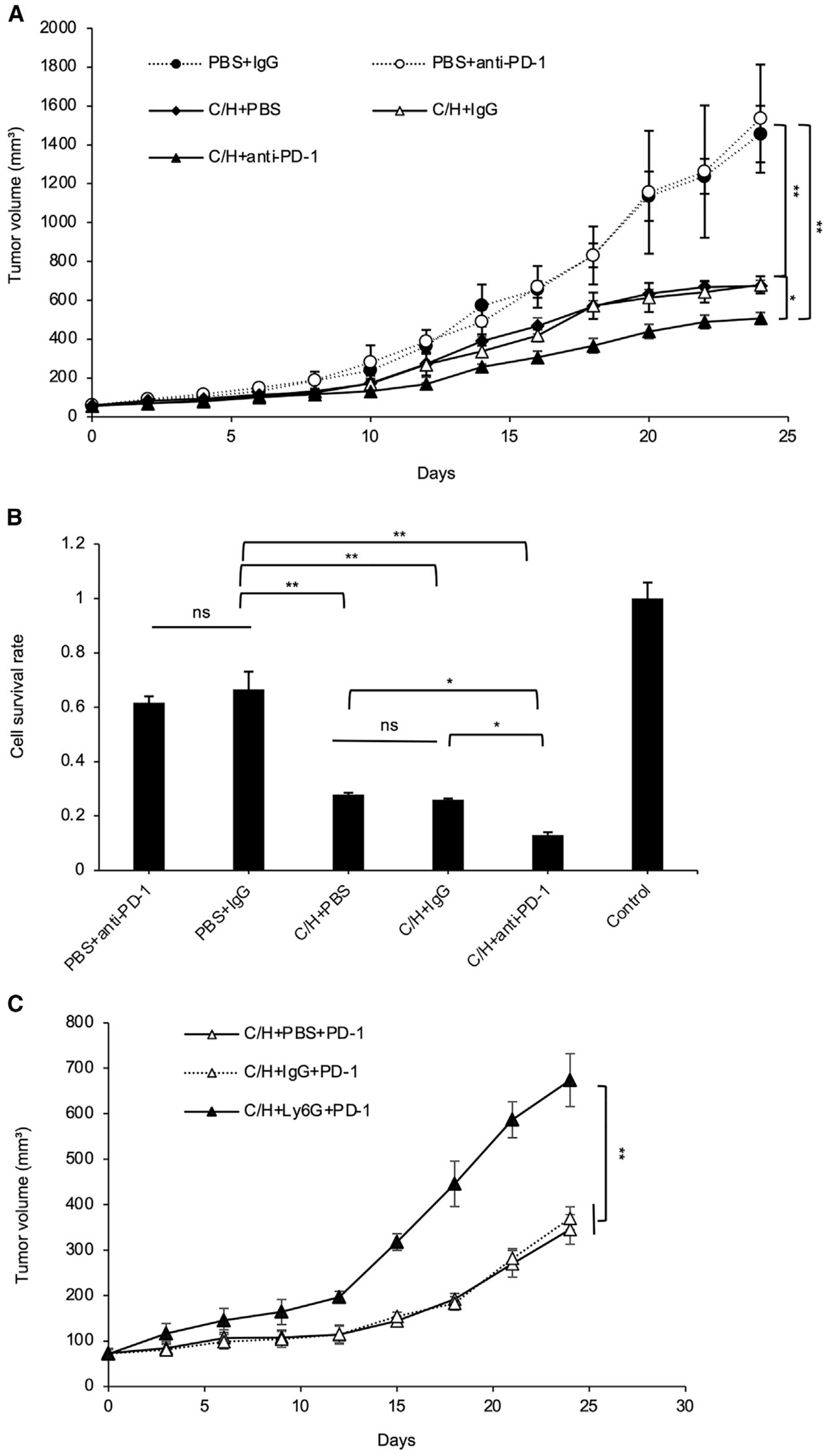


**Figure 6. C/H treatment improved the tumor suppression effect of anti-PD-1 antibody therapy in an orthotopic breast cancer model**

(A) 4T1 tumor-bearing mice were treated with an IT injection of PBS or C/H, followed by five injections of HVJ-E alone and an intraperitoneal injection of anti-PD-1 antibodies or control IgG. Tumor volumes were measured every other day, and the data are presented as the mean ± SD (n = 4 per group). (B) Mice from (A) were sacrificed 1 week after the final treatment to isolate $CD8^+$ T cells from the spleen, which were co-cultured with 4T1 cells at a 50:1 ratio for 24 h. Then, the killing activity of $CD8^+$ T cells was evaluated by 4T1 cell survival using an MTS assay. The data are shown as the mean ± SD (n = 4 per group). (C) To investigate the contribution of neutrophils to the enhancement of tumor suppression by combining C/H and anti-PD-1 antibodies, anti-Ly6G antibodies or control IgG were intraperitoneally administered to the mice treated with the combination therapy. Tumor volumes were measured every 2 days. The data are shown as the mean ± SD (n = 3 per group). *$p < 0.05$ and **$p < 0.01$. NS, not significant; Control, 4T1 without treatment.

reduced after C/H treatment compared with the other treatments. First, C/H may reduce the number of 4T1 cells scattering from the primary lesion. Second, CTLs activated by C/H may kill metastasized foci in the lungs. Our results showed that N1-type neutrophils increased in both the primary tumor mass and lungs (Figures 3A and 5B) and CTLs were activated systemically, because $CD8^+$ T cells from the spleen had tumor killing activity (Figure 3C, left). Thus, we speculate that C/H activated CTLs against cancer cells, which suppressed tumor growth in both the primary lesion and metastasized lung lesions.

Surprisingly, C/H treatment of the primary tumor mass increased N1-type neutrophils in the lung (Figure 5B). Both neutrophils and the N1 neutrophil population were decreased in total tumor and lung with C/H treatment using anti-Ly6G antibody (Figure S6). C/H treatment also decreased the fas− population in the tumors but not in the lungs (Figure S6). Although we have not observed neutrophil accumulation in other organs, N1-type neutrophils may have increased systemically. If that is possible, C/H might inhibit the systemic metastasis of cancer cells by activating CTLs in various organs. It is curious that HVJ-E alone increased N1-type neutrophils in the lung, while CXCL2 plasmid DNA failed to accumulate neutrophils in the lung even though both HVJ-E and CXCL2 plasmid DNA increased neutrophils in primary tumor masses. We confirmed that HVJ-E did not induce CXCL2 secretion from the tumor mass, as shown in Figure S2. One paper showed that CXCL1 can induce IL-17 secretion from the activation of $CD8^+$ T cells. Then, IL-17 induces $CD4^+$ T cells secreting CXCL2, which causes neutrophil recruitment.[44] We found that 4T1 cells produced CXCL1, and HVJ-E treatment enhanced the secretion of CXCL1 from 4T1 cells (data not shown). We also found C/H treatment can increase the population of $CD4^+$ T cells in the lungs (Figure S5B). Based on these results, we may suppose that IL-17 from activated $CD8^+$ T cells induces CXCL2 secretion from $CD4^+$ T cells, which contributes to neutrophil infiltration into metastatic lesions.

Because HVJ-E itself has various anti-tumor activities, clinical trials are ongoing on melanoma, prostate cancer, and malignant mesothelioma regarding the approval of HVJ-E as an anti-cancer biomedicine. The next step will be cancer gene therapy using HVJ-E. C/H will be the first candidate, and the triple combination with anti-PD-1 antibodies will become a cancer immunotherapy of great promise.

## MATERIALS AND METHODS

### Cell lines and mice

The 4T1 mammary carcinoma cell line was acquired from the American Type Culture Collection (Manassas, VA, USA), maintained in RPMI 1640 medium (Nacalai Tesque, Kyoto, Japan) with 10% fetal bovine serum (FBS) (BioWest, Nuaille, France) and 0.1 mg/mL penicillin-streptomycin (Nacalai Tesque), and incubated at 37°C in a humidified atmosphere of 5% $CO_2$. The 4T1-lucifrease cell line was received from the Japanese Collection of Research Bioresources (Osaka, Japan), and the BALB-MC.E12 cell line was obtained from the Japanese Collection of Research Bioresources Cell Bank (Osaka, Japan); the culture conditions were the same as those of the 4T1 mammary carcinoma cell line. No mycoplasma contamination was detected in any of the cell lines. Six- to eight-week-old female BALB/c mice (CLEA Japan, Tokyo, Japan) were housed in a temperature-controlled, pathogen-free room. All animal procedures were performed in accordance with the approved protocols and guidelines of the Animal Committee of Osaka University (Suita, Japan).

### Virus production and inactivation

HVJ (VR-105 parainfluenza Sendai/52 Z strain) was acquired from the American Type Culture Collection (Manassas, VA, USA) and prepared as previously described.[16] The HVJ seed solution was injected into embryonated eggs that were 10–14 days old and cultured in a 37°C incubator for 3 days. After 3 days, chorioallantoic fluid was harvested from the eggs injected with HVJ. The purified virus (live HVJ) was inactivated by UV irradiation (189 $mJ/cm^2$) to become HVJ-E.

### Tumor treatment

A total of $1 \times 10^6$ viable 4T1 breast cancer cells or BALB-MC.E12 mouse mammary tumor cells (in 50 μL of PBS) were intradermally injected into the backs (experiments in Figures 1 and 2) or the fourth mammary gland to create on orthotopic model (experiments in Figures 3, 4, and 6) using BALB/c mice. Four days later, when the tumor was 3–5 mm in diameter, the mice were IT injected once with HVJ-E (1,000 HAU), CXCL2 plasmid DNA (200 μg), or HVJ-E (1,000 HAU) combined with CXCL2 (40 μg or 200 μg) or pCY4B vector (200 μg) in 50 μL of PBS or with PBS (50 μL); they were then IT injected five times with HVJ-E (1,000 HAU) or PBS (50 μL) every other day. The tumor volume was measured in a blinded manner using slide calipers and was calculated using the following formula: tumor volume ($mm^3$) = length $\times$ $(width)^2$ /2.

### ELISpot assay

The 4T1 tumor-bearing mice were treated IT with CXCL2 plasmid DNA, HVJ-E, or CXCL2 in combination with HVJ-E once, followed by five additional treatments of HVJ-E every other day for a total of six treatments. The spleens were isolated from the mice 14 days after the last treatment. Splenocytes were isolated from the spleens, filtered through a 40-μm mesh sieve, and hemolyzed in hemolysis buffer (Immuno-Biological Laboratories). The 4T1 cells were treated with mitomycin C (15 μg/mL) for 45 min. The splenocytes and mitomycin C-treated 4T1 cells were mixed at a ratio of 10:1 and incubated at 37°C in a humidified atmosphere of 5% $CO_2$. After 48 h, nonadherent splenocytes were collected, and an ELISpot assay was performed using the Mouse IFN-gamma Development Module (R&D Systems, Minneapolis, MN, USA) and the ELISpot Blue Color Module (R&D Systems, Minneapolis, MN, USA). The numbers of IFN-gamma-secreting cells were subsequently counted.

### Flow cytometry analysis of the tumors and lungs

Tumors were collected from the mice and minced into fine pieces in a digestion buffer containing 2% FBS and 2.5 mg/mL collagenase A (Roche, Basel, Switzerland). The samples were incubated in the digestion buffer at 37°C for 1 h with a shaker, filtered through a 70-μm filter,

and washed twice with PBS. The lungs were collected from the mice and minced into fine pieces in a digestion buffer containing 2% FBS and 1.5 mg/mL collagenase B (Roche, Basel, Switzerland). The samples were incubated in the digestion buffer at 37°C for 45 min with a shaker, filtered through a 70-μm filter, hemolyzed in hemolysis buffer (Immuno-Biological Laboratories), and washed twice with PBS. The collected cells were stained with the following fluorescent labeled antibodies: CD45 (Clone: 30-F11, 103134, Biolegend, San Diego, CA, USA), CD11b (Clone: M1/70, 101216, Biolegend), Ly6G (Clone: 1A8, 127614, Biolegend), and FAS (Clone: Jo2, eBioscience, San Diego, CA, USA). All flow cytometry was performed on a BD FACSCanto II (Becton Dickinson, USA), and the analyses were performed using FlowJo software (FlowJo, Ashland, OR, USA).

#### Anti-Ly6G antibodies and CXCL2 plasmid DNA in combination with HVJ-E 4T1 tumor model mouse treatment

For the neutrophil-blocking experiment, 4T1 tumor-bearing mice were pretreated with an intraperitoneal injection of Ultra-LEAF Purified anti-mouse Ly-6G antibodies (100 μg in 50 μL of PBS, 1A8, 127649, Biolegend) or IgG from rat serum (100 μg in 50 μL of PBS, 14131, Sigma-Aldrich, Japan) six times 24 h before being IT injected with CXCL2 plasmid DNA (200 μg in 50 μL of PBS), HVJ-E (1,000 HAU in 50 μL of PBS), CXCL2 in combination with HVJ-E (200 μg and 1,000 HAU in 50 μL of PBS), or PBS (50 μL) once, followed by five additional treatments of HVJ-E (1,000 HAU in 50 μL of PBS) or PBS (50 μL) treatment. After the final injection, the tumor size was measured every 2 days.

#### CTL activation experiment by MTS assays

Cell viability was determined using the Cell Titer 96 Aqueous One Solution Cell Proliferation Assay kit (Promega, WI, USA). Briefly, after treatment, $CD8^+$ T cells were isolated from all the treated mice splenocytes using the Mojo Sort mouse CD8 T cell Isolation Kit (480035, Biolegend) following the manufacturer's protocol. The isolated $CD8^+$ T cells ($5 \times 10^4$ cells in 50 μL of culture medium/well) were co-cultured with 4T1 cells at a ratio of 50:1 and incubated at 37°C in a humidified atmosphere of 5% $CO_2$. After 24 h, 20 μL of Cell Titer 96 Aqueous One Solution reagent was added to each well, and the plates were incubated at 37°C in 5% $CO_2$ for 2 h. After transferring 100 μL of incubation medium from each well into a new 96-well plate, the absorbance was measured at 490 nm.

#### Immunostaining of immune cells in 4T1 lung tissues

After the 4T1 cancer cell mouse model was treated with CXCL2 plasmid DNA (pDNA) in combination with HVJ-E treatment, HVJ-E treatment, CXCL2 pDNA treatment, or PBS treatment for 3 weeks, lung sections were fixed with 4% paraformaldehyde solution and blocked with 5% BSA. The sections were stained with Ultra-LEAF Purified anti-mouse Ly-6G antibodies (1A8, 127649, Biolegend). The secondary antibodies included an Alexa Fluor 488-conjugated rabbit anti-rat IgG (Life Technologies, Carlsbad, CA, USA). The sections were mounted in Vectashield mounting medium (Vector Laboratories, Burlingame, CA, USA) and imaged with a confocal laser scanning microscope (LSM880 with Airyscan, Zeiss, Jena, Germany) equipped with the ZEN software program.

#### Luciferase assay

A luciferase assay was performed after 1 week of treatment. The lungs were harvested from 4T1 tumor-bearing mice and minced into fine pieces in a digestion buffer containing 2% FBS and 1.5 mg/mL collagenase B (Roche, Basel, Switzerland). The samples were incubated in the digestion buffer at 37°C for 45 min with a shaker, filtered through a 70-μm filter, hemolyzed in hemolysis buffer (Immuno-Biological Laboratories), and washed twice with PBS. The collected cells were analyzed with a Luciferase Assay System (Promega, Fitchburg, WI, USA) following the manufacturer's protocol. A 96-well Mithras LB 940 Multimode Microplate Reader (Berthold Technologies, Bad Wildbad, Germany) was used to measure the result with a luminescence program.

#### Anti-PD-1 antibodies and CXCL2 plasmid DNA in combination with HVJ-E 4T1 tumor model mouse treatment

A total of $5 \times 10^5$ viable 4T1 breast cancer cells (in 50 μL of PBS) were intradermally injected into the right fourth mammary gland of BALB/c mice. Tumor-bearing mice were treated with an intraperitoneal injection of InVivoPlus anti-mouse PD-1 (CD279) (250 μg in 50 μL of PBS, RMP1-14, BP0146, BioXcell), IgG from rat serum (250 μg in 50 μL of PBS, 14131, Sigma-Aldrich, Japan), or PBS (50 μL) and IT injected with C/H or PBS (50 μL), followed by treatments of HVJ-E (1,000 HAU in 50 μL of PBS) or PBS (50 μL). The tumor volume was measured every 2 days.

#### Anti-Ly6G antibody neutralization of neutrophils in C/H plus anti-PD-1 antibody treatment

A total of $1 \times 10^6$ viable 4T1 breast cancer cells (in 50 μL of PBS) were intradermally injected into the right fourth mammary gland of BALB/c mice. Tumor-bearing mice were pretreated with an intraperitoneal injection of Ultra-LEAF Purified anti-mouse Ly-6G antibodies (100 μg in 50 μL of PBS, 1A8, 127649, Biolegend), IgG from rat serum (100 μg in 50 μL of PBS, 14131, Sigma-Aldrich, Japan), or PBS (50 μL) 24 h before tumor treatment. Then, the mice were treated with an intraperitoneal injection of InVivoPlus anti-mouse PD-1 (CD279) (250 μg in 50 μL of PBS, RMP1-14, BP0146, BioXcell), control IgG from rat serum (250 μg in 50 μL of PBS, 14131, Sigma-Aldrich, Japan), or PBS (50 μL) and one IT injection of C/H, followed by HVJ-E (200 μg and 1,000 HAU in 50 μL of PBS) or PBS (50 μL), followed by treatments of HVJ-E (1,000 HAU in 50 μL of PBS) or PBS (50 μL) treatment. The tumor volume was measured every 2 days.

#### Statistical analysis

The results are shown as the mean ± SD. The statistical analysis was performed using Prism GraphPad 9.0 and Microsoft office Excel. Differences between two groups were evaluated by two-tailed Student's t tests, multiple groups were compared with one-way analysis of variance (ANOVA), and tumor volume groups were assessed by the Tukey-Kramer test. Results were considered

statistically significant when *p < 0.05, **p < 0.01, ***p < 0.001, and ****p < 0.0001.

## SUPPLEMENTAL INFORMATION

Supplemental Information can be found online at https://doi.org/10.1016/j.omto.2020.12.011.

## ACKNOWLEDGMENTS

This work has been supported by grants to Y.K. from the Japan Agency for Medical Research and Development (17lm0203092h0001, 18lm0203092h0001, and 19lm0203092h0001) from 2017–2020.

## AUTHOR CONTRIBUTIONS

T.N. and Y. K. designed the experiments. Y.C.P. performed the experiments, analyzed the data, and wrote the paper. T.N., C.Y.C., J.A.T., and Y.K. made suggestions for the manuscript.

## DECLARATION OF INTERESTS

The Department of Device Application for Molecular Therapeutics is a joint research department supported by Daicel, Co. The authors declare no competing interests.

## REFERENCES


1. Maajani, K., Jalali, A., Alipour, S., Khodadost, M., Tohidinik, H.R., and Yazdani, K. (2019). The Global and Regional Survival Rate of Women With Breast Cancer: A Systematic Review and Meta-analysis. Clin. Breast Cancer *19*, 165–177.
2. Homet Moreno, B., and Ribas, A. (2015). Anti-programmed cell death protein-1/ligand-1 therapy in different cancers. Br. J. Cancer *112*, 1421–1427.
3. Tumeh, P.C., Harview, C.L., Yearley, J.H., Shintaku, I.P., Taylor, E.J., Robert, L., Chmielowski, B., Spasic, M., Henry, G., Ciobanu, V., et al. (2014). PD-1 blockade induces responses by inhibiting adaptive immune resistance. Nature *515*, 568–571.
4. Meng, X., Huang, Z., Teng, F., Xing, L., and Yu, J. (2015). Predictive biomarkers in PD-1/PD-L1 checkpoint blockade immunotherapy. Cancer Treat. Rev. *41*, 868–876.
5. Chowdhury, P.S., Chamoto, K., and Honjo, T. (2018). Combination therapy strategies for improving PD-1 blockade efficacy: a new era in cancer immunotherapy. J. Intern. Med. *283*, 110–120.
6. Beckermann, K.E., Johnson, D.B., and Sosman, J.A. (2017). PD-1/PD-L1 blockade in renal cell cancer. Expert Rev. Clin. Immunol. *13*, 77–84.
7. Ansell, S.M., Lesokhin, A.M., Borrello, I., Halwani, A., Scott, E.C., Gutierrez, M., Schuster, S.J., Millenson, M.M., Cattry, D., Freeman, G.J., et al. (2015). PD-1 blockade with nivolumab in relapsed or refractory Hodgkin's lymphoma. N. Engl. J. Med. *372*, 311–319.
8. Gravelle, P., Burroni, B., Péricart, S., Rossi, C., Bezombes, C., Tosolini, M., Damotte, D., Brousset, P., Fournié, J.J., and Laurent, C. (2017). Mechanisms of PD-1/PD-L1 expression and prognostic relevance in non-Hodgkin lymphoma: a summary of immunohistochemical studies. Oncotarget *8*, 44960–44975.
9. Imbert, C., Montfort, A., Fraisse, M., Marcheteau, E., Gilhodes, J., Martin, E., Bertrand, F., Marcellin, M., Burlet-Schiltz, O., Peredo, A.G., et al. (2020). Resistance of melanoma to immune checkpoint inhibitors is overcome by targeting the sphingosine kinase-1. Nat. Commun. *11*, 437.
10. Bertucci, F., and Gonçalves, A. (2017). Immunotherapy in Breast Cancer: the Emerging Role of PD-1 and PD-L1. Curr. Oncol. Rep. *19*, 64.
11. Kaneda, Y. (2013). The RIG-I/MAVS signaling pathway in cancer cell-selective apoptosis. OncoImmunology *2*, e23566.
12. Liu, L.W., Nishikawa, T., and Kaneda, Y. (2016). An RNA Molecule Derived From Sendai Virus DI Particles Induces Antitumor Immunity and Cancer Cell-selective Apoptosis. Mol. Ther. *24*, 135–145.
13. Kurooka, M., and Kaneda, Y. (2007). Inactivated Sendai virus particles eradicate tumors by inducing immune responses through blocking regulatory T cells. Cancer Res. *67*, 227–236.
14. Saga, K., and Kaneda, Y. (2013). Virosome presents multimodel cancer therapy without viral replication. BioMed Res. Int. *2013*, 764706.
15. Saga, K., Tamai, K., Yamazaki, T., and Kaneda, Y. (2013). Systemic administration of a novel immune-stimulatory pseudovirion suppresses lung metastatic melanoma by regionally enhancing IFN-γ production. Clin. Cancer Res. *19*, 668–679.
16. Chang, C.Y., Tai, J.A., Li, S., Nishikawa, T., and Kaneda, Y. (2016). Virus-stimulated neutrophils in the tumor microenvironment enhance T cell-mediated anti-tumor immunity. Oncotarget *7*, 42195–42207.
17. De Filippo, K., Dudeck, A., Hasenberg, M., Nye, E., van Rooijen, N., Hartmann, K., Gunzer, M., Roers, A., and Hogg, N. (2013). Mast cell and macrophage chemokines CXCL1/CXCL2 control the early stage of neutrophil recruitment during tissue inflammation. Blood *121*, 4930–4937.
18. Rouault, C., Pellegrinelli, V., Schilch, R., Cotillard, A., Poitou, C., Tordjman, J., Sell, H., Clément, K., and Lacasa, D. (2013). Roles of chemokine ligand-2 (CXCL2) and neutrophils in influencing endothelial cell function and inflammation of human adipose tissue. Endocrinology *154*, 1069–1079.
19. Brandau, S., Dumitru, C.A., and Lang, S. (2013). Protumor and antitumor functions of neutrophil granulocytes. Semin. Immunopathol. *35*, 163–176.
20. Carus, A., Ladekarl, M., Hager, H., Pilegaard, H., Nielsen, P.S., and Donskov, F. (2013). Tumor-associated neutrophils and macrophages in non-small cell lung cancer: no immediate impact on patient outcome. Lung Cancer *81*, 130–137.
21. Uribe-Querol, E., and Rosales, C. (2015). Neutrophils in Cancer: Two Sides of the Same Coin. J. Immunol. Res. *2015*, 983698.
22. Biswas, S.K., Sica, A., and Lewis, C.E. (2008). Plasticity of macrophage function during tumor progression: regulation by distinct molecular mechanisms. J. Immunol. *180*, 2011–2017.
23. Lewis, C.E., and Pollard, J.W. (2006). Distinct role of macrophages in different tumor microenvironments. Cancer Res. *66*, 605–612.
24. Martinez, F.O., Helming, L., and Gordon, S. (2009). Alternative activation of macrophages: an immunologic functional perspective. Annu. Rev. Immunol. *27*, 451–483.
25. Mishalian, I., Bayuh, R., Levy, L., Zolotarov, L., Michaeli, J., and Fridlender, Z.G. (2013). Tumor-associated neutrophils (TAN) develop pro-tumorigenic properties during tumor progression. Cancer Immunol. Immunother. *62*, 1745–1756.
26. Fridlender, Z.G., Sun, J., Kim, S., Kapoor, V., Cheng, G., Ling, L., Worthen, G.S., and Albelda, S.M. (2009). Polarization of tumor-associated neutrophil phenotype by TGF-beta: "N1" versus "N2" TAN. Cancer Cell *16*, 183–194.
27. Dumitru, C.A., Lang, S., and Brandau, S. (2013). Modulation of neutrophil granulocytes in the tumor microenvironment: mechanisms and consequences for tumor progression. Semin. Cancer Biol. *23*, 141–148.
28. Eruslanov, E.B., Bhojnagarwala, P.S., Quatromoni, J.G., Stephen, T.L., Ranganathan, A., Deshpande, C., Akimova, T., Vachani, A., Litzky, L., Hancock, W.W., et al. (2014). Tumor-associated neutrophils stimulate T cell responses in early-stage human lung cancer. J. Clin. Invest. *124*, 5466–5480.
29. Granot, Z., Henke, E., Comen, E.A., King, T.A., Norton, L., and Benezra, R. (2011). Tumor entrained neutrophils inhibit seeding in the premetastatic lung. Cancer Cell *20*, 300–314.
30. Kaneda, Y., Nakajima, T., Nishikawa, T., Yamamoto, S., Ikegami, H., Suzuki, N., Nakamura, H., Morishita, R., and Kotani, H. (2002). Hemagglutinating virus of Japan (HVJ) envelope vector as a versatile gene delivery system. Mol. Ther. *6*, 219–226.
31. Li, J.L., Lim, C.H., Tay, F.W., Goh, C.C., Devi, S., Malleret, B., Lee, B., Bakocevic, N., Chong, S.Z., Evrard, M., et al. (2016). Neutrophils Self-Regulate Immune Complex-Mediated Cutaneous Inflammation through CXCL2. J. Invest. Dermatol. *136*, 416–424.
32. Fantozzi, A., and Christofori, G. (2006). Mouse models of breast cancer metastasis. Breast Cancer Res. *8*, 212.
33. Weigelt, B., Peterse, J.L., and van 't Veer, L.J. (2005). Breast cancer metastasis: markers and models. Nat. Rev. Cancer *5*, 591–602.

34. Malla, R.R., Deepak, K., Merchant, N., and Dasari, V.R. (2020). Breast Tumor Microenvironment: Emerging target of therapeutic phytochemicals. Phytomedicine *70*, 153227.

35. Nowicki, T.S., Hu-Lieskovan, S., and Ribas, A. (2018). Mechanisms of Resistance to PD-1 and PD-L1 Blockade. Cancer J. *24*, 47–53.

36. Wolchok, J.D., Kluger, H., Callahan, M.K., Postow, M.A., Rizvi, N.A., Lesokhin, A.M., Segal, N.H., Ariyan, C.E., Gordon, R.A., Reed, K., et al. (2013). Nivolumab plus ipilimumab in advanced melanoma. N. Engl. J. Med. *369*, 122–133.

37. Spiers, L., Coupe, N., and Payne, M. (2019). Toxicities associated with checkpoint inhibitors-an overview. Rheumatology (Oxford) *58* (*Suppl 7*), vii7–vii16.

38. Trujillo, J.A., Sweis, R.F., Bao, R., and Luke, J.J. (2018). T Cell-Inflamed versus Non-T Cell-Inflamed Tumors: A Conceptual Framework for Cancer Immunotherapy Drug Development and Combination Therapy Selection. Cancer Immunol. Res. *6*, 990–1000.

39. Planes-Laine, G., Rochigneux, P., Bertucci, F., Chrétien, A.S., Viens, P., Sabatier, R., and Gonçalves, A. (2019). PD-1/PD-L1 Targeting in Breast Cancer: The First Clinical Evidences Are Emerging. A Literature Review. Cancers (Basel) *11*, 1033.

40. Plitas, G., Konopacki, C., Wu, K., Bos, P.D., Morrow, M., Putintseva, E.V., Chudakov, D.M., and Rudensky, A.Y. (2016). Regulatory T Cells Exhibit Distinct Features in Human Breast Cancer. Immunity *45*, 1122–1134.

41. Fujihara, A., Kurooka, M., Miki, T., and Kaneda, Y. (2008). Intratumoral injection of inactivated Sendai virus particles elicits strong antitumor activity by enhancing local CXCL10 expression and systemic NK cell activation. Cancer Immunol. Immunother. *57*, 73–84.

42. Takeda, S., Terazono, B., Mishima, F., Nakagami, H., Nishijima, S., and Kaneda, Y. (2006). Novel drug delivery system by surface modified magnetic nanoparticles. J. Nanosci. Nanotechnol. *6*, 3269–3276.

43. Fujita, K., Nakai, Y., Kawashima, A., Ujike, T., Nagahara, A., Nakajima, T., Inoue, T., Lee, C.M., Uemura, M., Miyagawa, Y., et al. (2017). Phase I/II clinical trial to assess safety and efficacy of intratumoral and subcutaneous injection of HVJ-E in castration-resistant prostate cancer patients. Cancer Gene Ther. *24*, 277–281.

44. Jin, L., Batra, S., Douda, D.N., Palaniyar, N., and Jeyaseelan, S. (2014). CXCL1 contributes to host defense in polymicrobial sepsis via modulating T cell and neutrophil functions. J. Immunol. *193*, 3549–3558.

**OMTO, Volume *20***

## Supplemental Information

# CXCL2 combined with HVJ-E suppresses tumor growth and lung metastasis in breast cancer and enhances anti-PD-1 antibody therapy

**Yi Chun Pan, Tomoyuki Nishikawa, Chin Yang Chang, Jiayu A. Tai, and Yasufumi Kaneda**

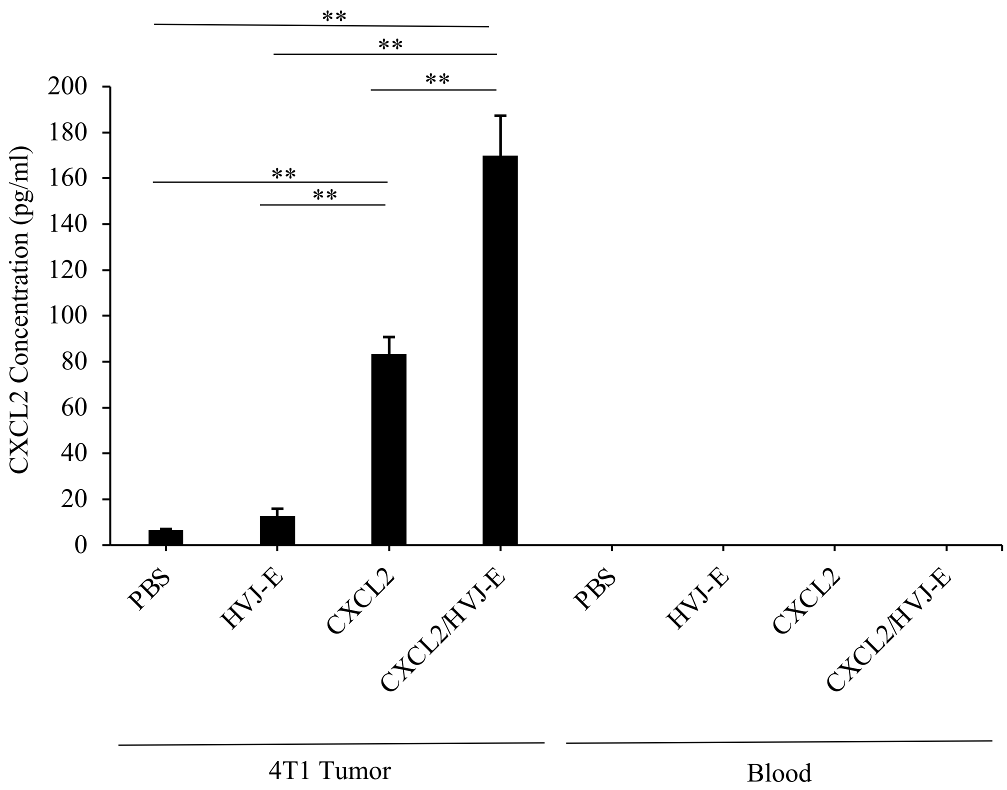


Figure. S1 CXCL2 in combination with HVJ-E increased CXCL2 in 4T1 tumor

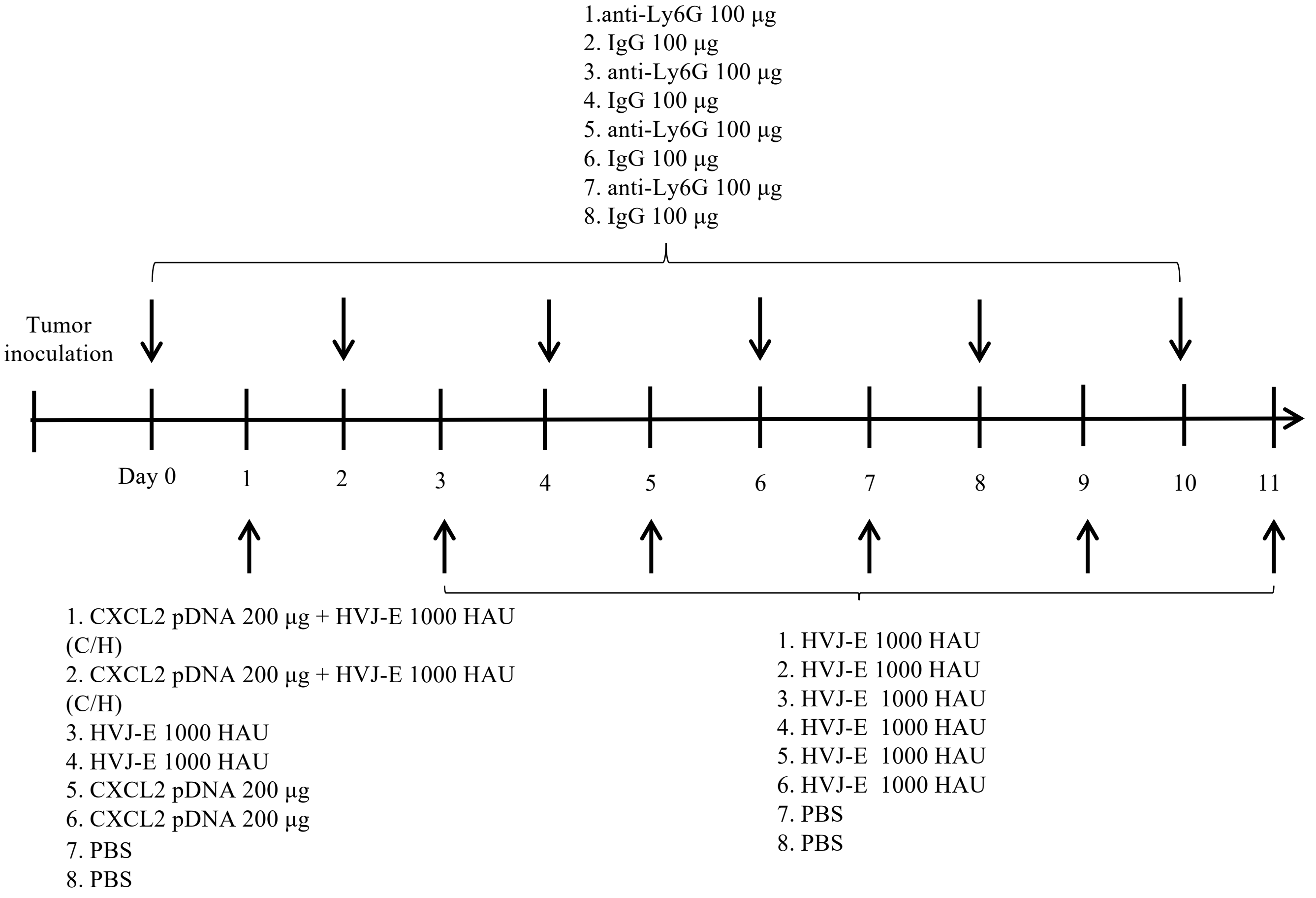


Figure. S2 The protocol for each treatment

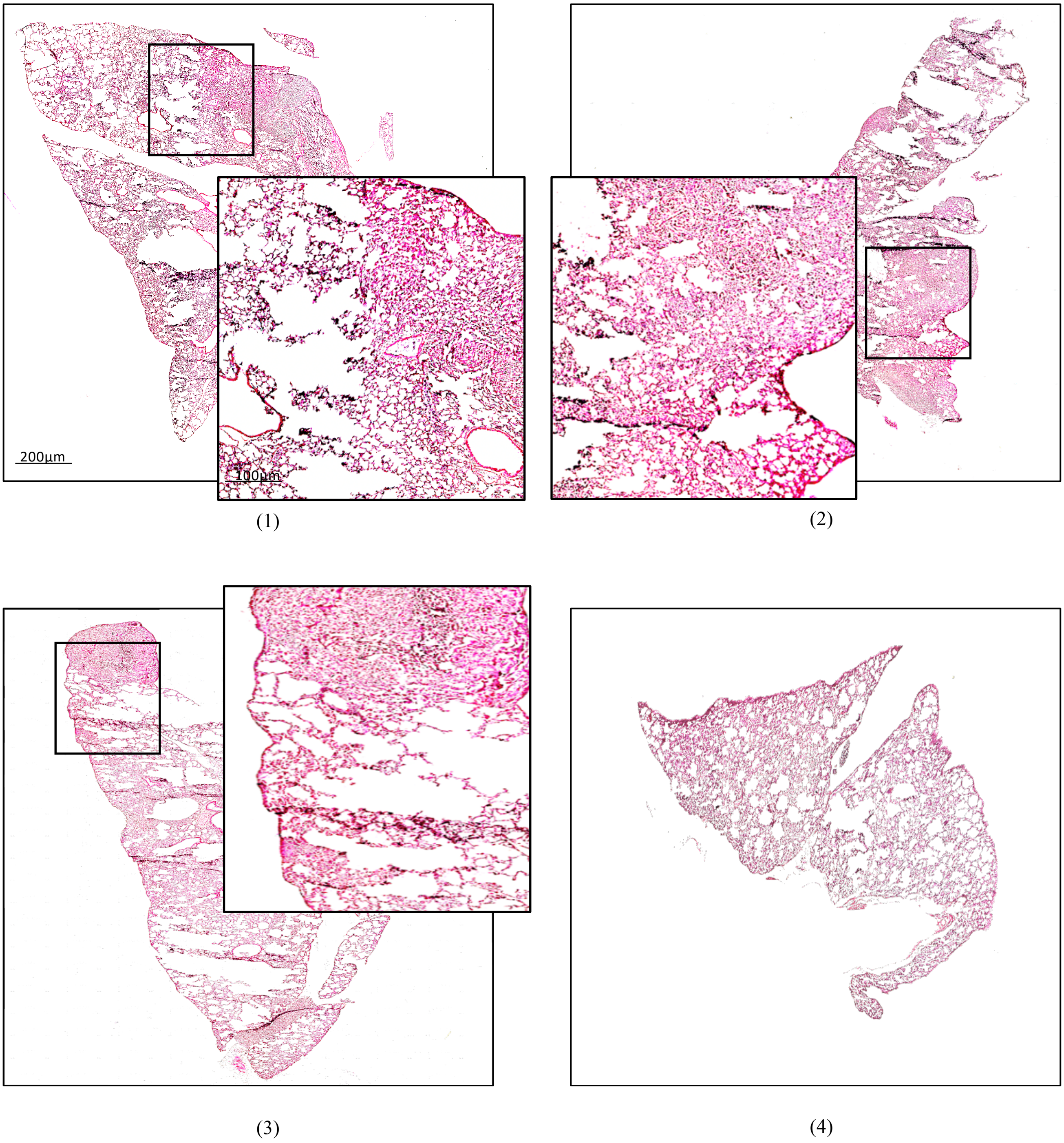


Figure.S3 C/H treatment suppressed 4T1 tumor metastasis to mouse lung

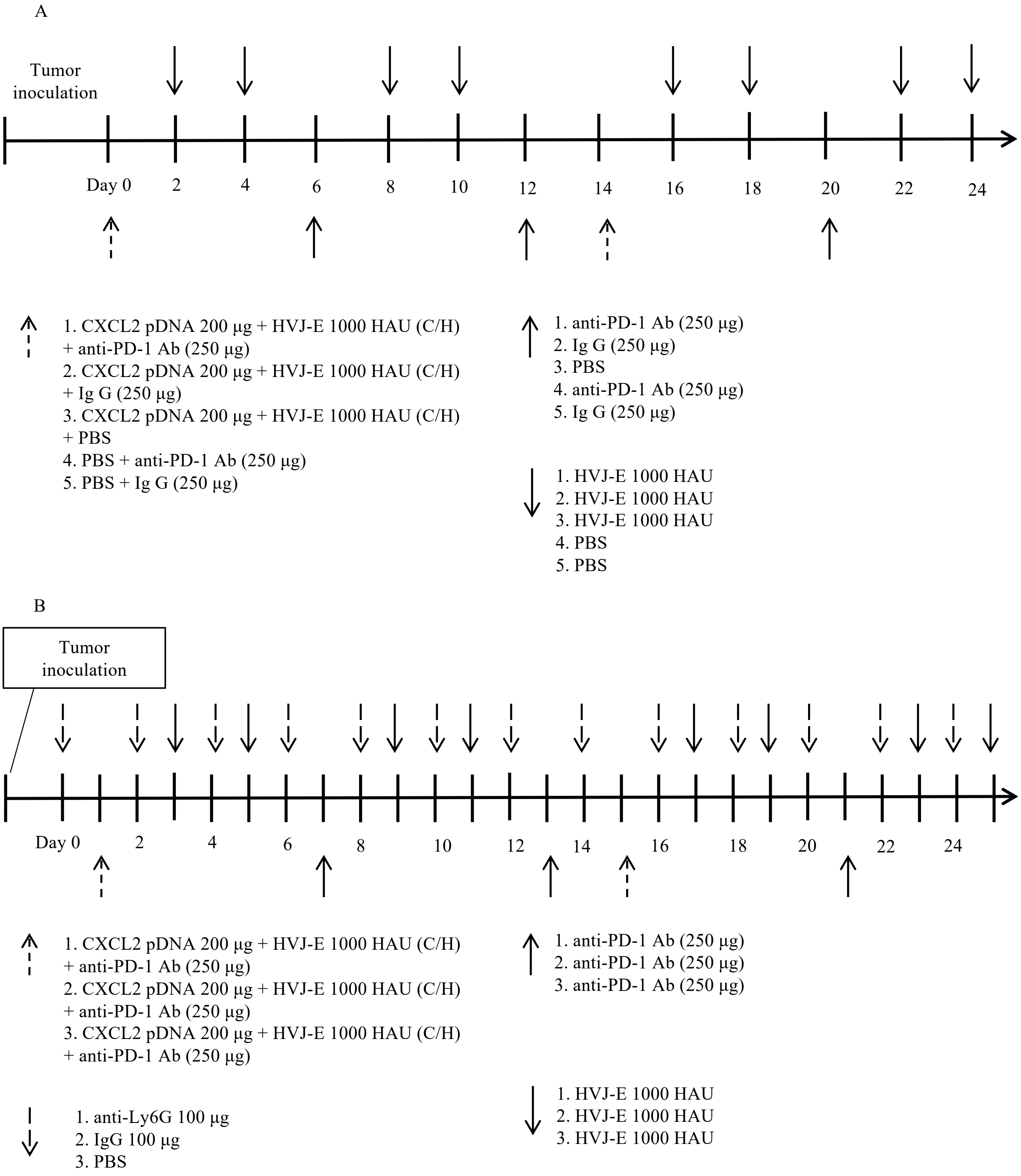


Figure. S4 The schedule of tumor treatment

A 4T1 Tumor

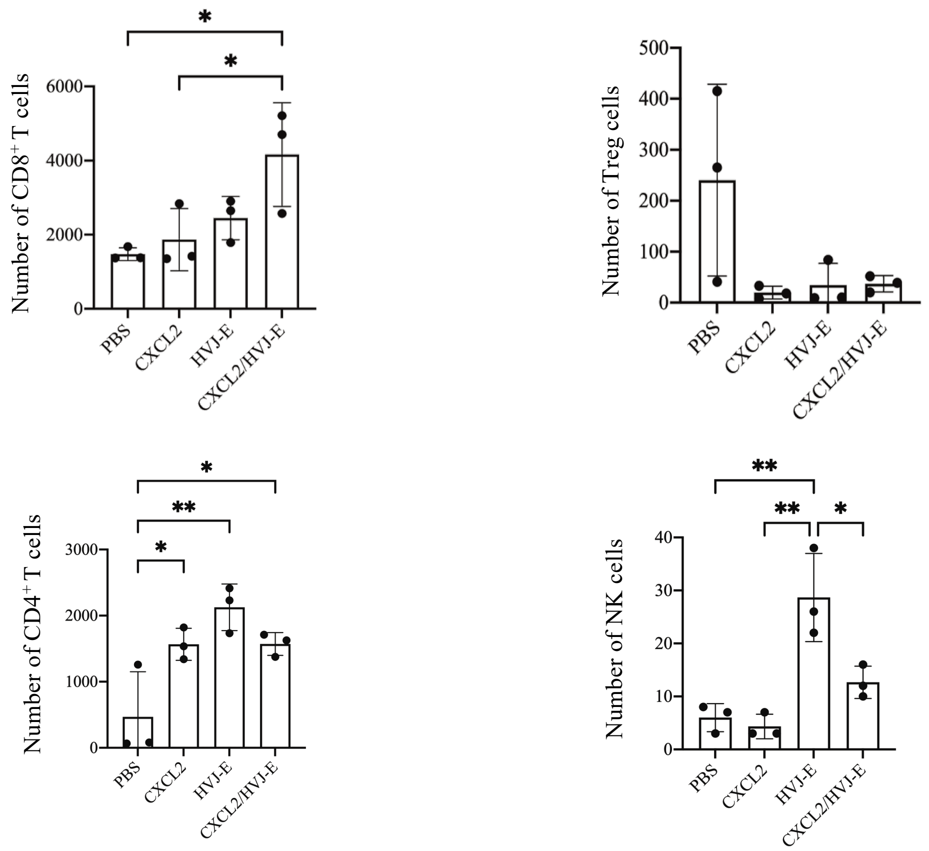


B 4T1 Lung

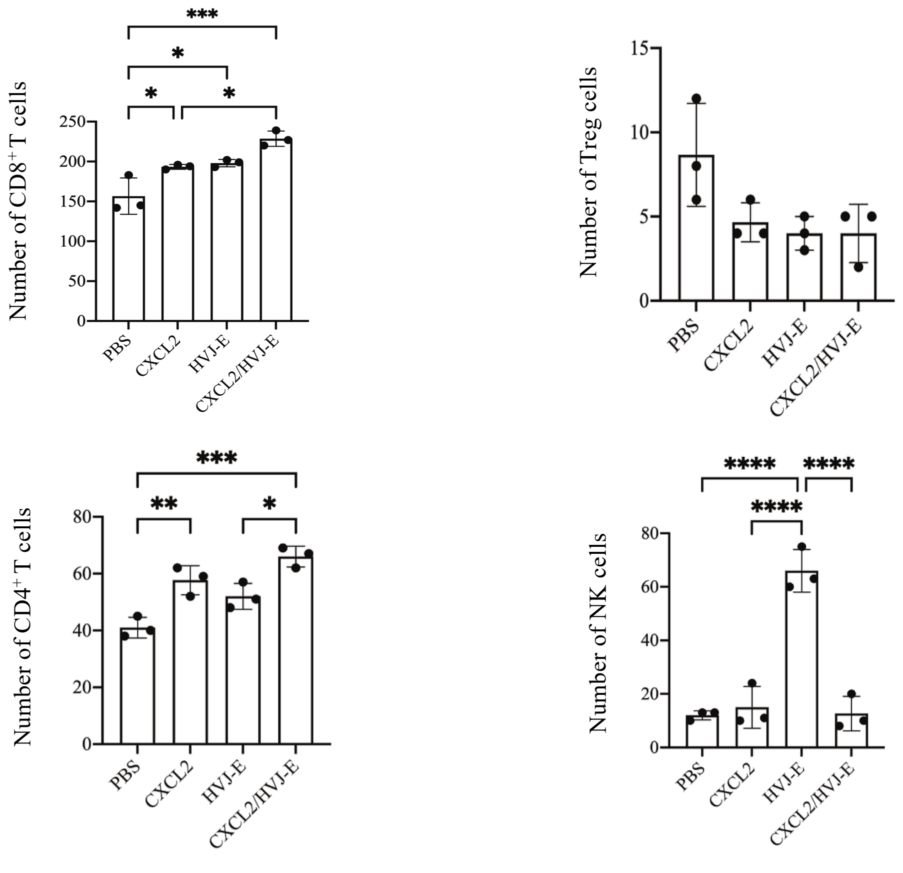


Figure. S5 C/H treatment induced CD8+ T cells in tumor and lung.

4T1 Tumor

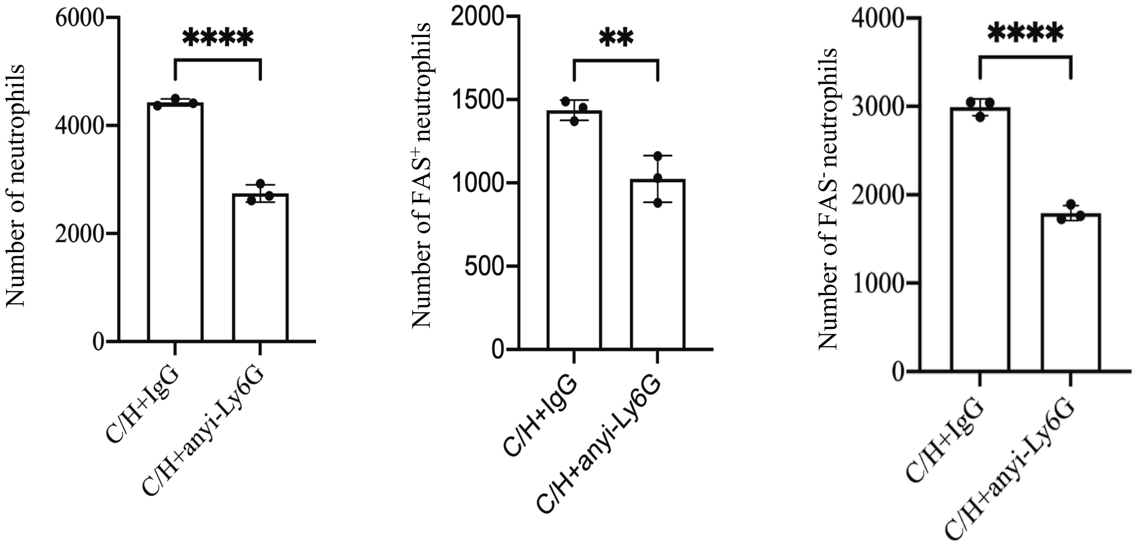


4T1 Lung

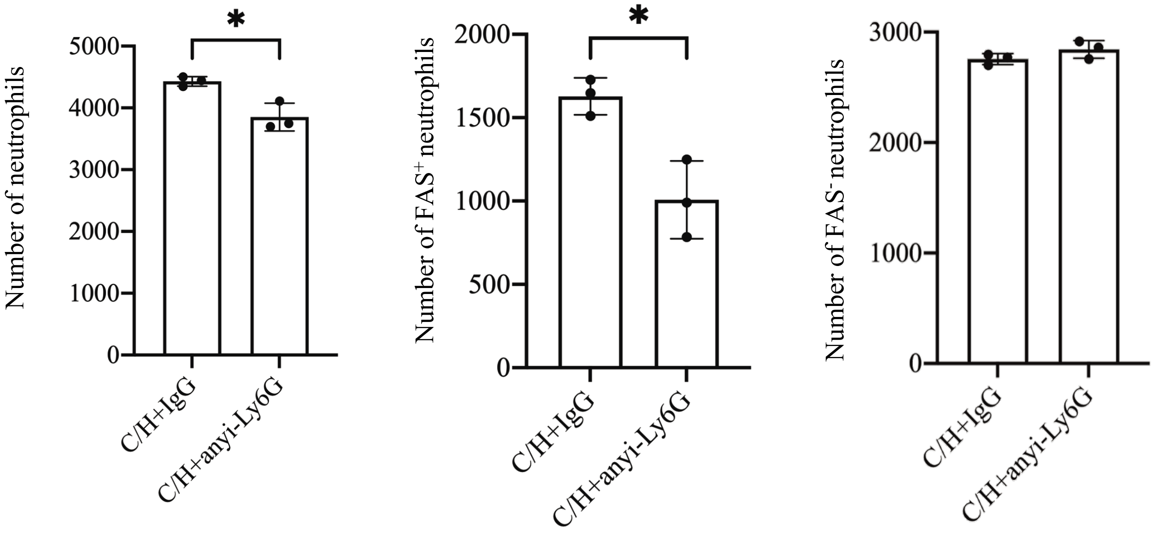


Figure. S6 The influence on C/H treatment with anti-Ly6G in 4T1 tumor and lung.

A

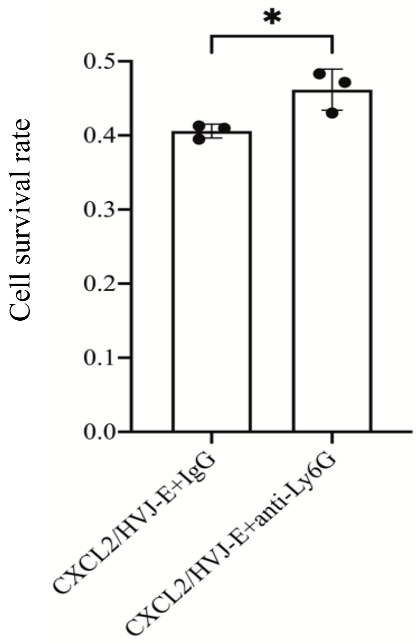


B

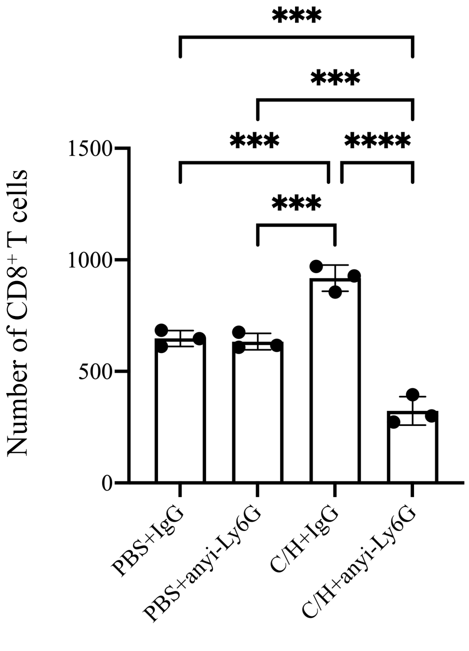


Figure. S7 **C/H treatment increased CD8$^{+}$ T cells and induced CTLs activation.**

Figure. S1

**CXCL2 in combination with HVJ-E increased CXCL2 in 4T1 tumor.** The expression of CXCL2 in 4T1 tumors treated with PBS, HVJ-E, CXCL2 plasmid DNA, or C/H was measured with a CXCL2 ELISA. The CXCL2 protein level in the blood of 4T1 tumor-bearing mice was also measured. Mouse model measured by an ELISA assay. The data are shown as the mean ± SD (n = 4 per group). ** and * indicate $p < 0.01$ and $p < 0.05$, respectively.

Figure. S2

**The protocol for each treatment.** The protocol for each treatment is shown. The schedule of anti-Ly6G antibody administration in the 4T1 tumor mouse model is also shown.

Figure. S3

**C/H treatment suppressed 4T1 tumor metastasis in the mouse lung.** After each treatment, 4T1 tumor-bearing mice were sacrificed, and the lungs were analyzed by HE staining. (1), (2), (3), and (4) indicate the PBS treatment, CXCL2 plasmid DNA treatment, HVJ-E treatment, and C/H treatment, respectively. Lung tissues with metastasizing foci were magnified in (1), (2) and (3).

Figure. S4

**The tumor treatment schedule.** (A) The schedule of C/H treatment combined with anti-PD-1 antibodies in a 4T1 tumor mouse model is described. (B) The schedule of anti-Ly6G antibody administration on C/H treatment combined with anti-PD-1 antibodies in a 4T1 tumor mouse model is described.

Figure. S5

**The number of immune cells in 4T1 primary and lung metastatic lesions.** (A) The number of CD8+ T cells, CD4+ T cells, Treg cells and NK cells in tumors 24 hours after the final treatment were measured by flow cytometry. (B) The number of CD8+ T cells, CD4+ T cells, Treg cells ($CD4^+$, $CD25^+$) and NK cells ($CD49b^+$, $NKp46^+$) in lungs 1 week after the final treatment were measured by flow cytometry. The mean ± SD (n = 3 per group) was shown. ∗, ∗∗, ∗∗∗ and ∗∗∗∗ indicate $p < 0.05$, $p < 0.01$, $p < 0.001$ and $p < 0.0001$, respectively.

Figure. S6

**The influence of anti-Ly6G antibody on neutrophil infiltration in 4T1 primary and lung metastatic lesions treated with C/H.** The number of neutrophils ($CD11b^+$, $Ly\text{-}6G^+$), $Fas^+$ neutrophils ($CD11b^+$, $Ly\text{-}6G^+$, $Fas^+$) and $Fas^-$ neutrophils ($CD11b^+$, $Ly\text{-}6G^+$, $Fas^-$) in 4T1 tumors (upper figures) and lungs (under figures) 24 hours or 1 week after the C/H treatment with anti-Ly6G or control IgG were analyzed by flow cytometry. ∗, ∗∗ and ∗∗∗∗ indicate $p < 0.05$, $p < 0.01$and $p < 0.0001$, respectively.

Figure. S7

**C/H treatment increased $CD8^+$ T cells and induced CTLs activation.** (A) The effect of anti-Ly6G antibody administration on $CD8^+$ T cell-mediated cell killing was compared with that of control IgG administration in 4T1 tumor model treated with C/H. (B) The number of CD8+ T cells in tumors 24 hours after PBS treatment and C/H treatment using anti-Ly6G or control IgG were measured by flow cytometry. The mean ± SD (n = 3 per group). ∗ and ∗∗∗ indicate $p < 0.05$ and $p < 0.001$, respectively.

**Supplemental Materials and Methods**

**Analysis of CXCL2 protein expression of tumor tissues and blood by CXCL2 ELISA assay**

Tumor tissues and blood were collected from 4T1 tumor-bearing mice 48 hours after the final treatment. The collected tissues were submerged in RIPA buffer (Nacalai Tesque Inc., Kyoto, Japan) at 2500 rpm for 20 seconds using a Multi-Beads Shocker (Yasui Kikai Co. Osaka, Japan). After homogenization on ice for 1 hour, the lysate was centrifuged at 15,000 x rpm for 10 minutes. The samples were frozen at -80° C. The collected blood was maintained at room temperature for 1 hour and centrifuged at 12 x G for 10 minutes. The serum was saved and frozen at -80° C. The samples were thawed and centrifuged at 10,000 x G for 5 minutes to remove cell debris. Tissue lysates containing equal amounts of protein (400 μg) and serum (25 μl) were subsequently used. The CXCL2 ELISA assay used a Mouse CXCL2 ELISA kit (MM200, R&D Systems, Minneapolis, USA) depending on the maker's protocol. A 96-well Mithras LB 940 Multimode Microplate Reader (Berthold Technologies GmbH & Co. KG, Bad Wildbad, Germany) was used to measure the result at 540 nm.

**H&E staining**

After the 4T1-bearing mouse model was treated with the C/H, HVJ-E, CXCL2 pDNA, or PBS treatments for three weeks, lung sections were fixed with 4% paraformaldehyde solution, embedded in OCT compound (Sakura Finetek Japan Co., Ltd., Tokyo, Japan), and sectioned at -80° C. Histological examinations were performed based on HE staining. HE staining procedures were subsequently performed on dehydrated sections and observed using a microscope (BZ-X700; Keyence, Osaka, Japan).

**Supplemental Materials and Methods**

**Flow cytometry analysis of the tumors and lungs**

Tumors were collected from the mice and minced into fine pieces in a digestion buffer containing 2% FBS and 2.5 mg/ml collagenase A (Roche, Basel, Switzerland). The samples were incubated in the digestion buffer at 37° C for 1 hour with a shaker, filtered through a 70-μm filter, and washed twice with PBS. The lungs were collected from the mice and minced into fine pieces in a digestion buffer containing 2% FBS and 1.5 mg/ml collagenase B (Roche, Basel, Switzerland). The samples were incubated in the digestion buffer at 37° C for 45 min with a shaker, filtered through a 70-μm filter, hemolyzed in hemolysis buffer (Immuno-Biological Laboratories Co., Ltd.), and washed twice with PBS. The collected cells were stained with the following fluorescent labeled antibodies: CD45 (Clone: 30-F11, 103134, Biolegend, Inc., San Diego, CA), CD3 (Clone: 17A2, 100236, Biolegend), CD8 (Clone: 53-6.7, 100726, Biolegend), CD4 (Clone: RM4-5, 100509, Biolegend), CD25 (Clone: 3C7, 101915, Biolegend), CD49b (Clone: DX5, 108907, Biolegend), NKp46 (Clone: 29A1.4, 137607, Biolegend), CD11b (Clone: M1/70, 101216, Biolegend), Ly6G (Clone: 1A8, 127614, Biolegend), and FAS (Clone: Jo2, eBioscience, San Diego, USA). All flow cytometry was performed on a Cyto FLEX S System B2-R3-V4-Y4 (BECKMAN COULTER, USA), and the analyses were performed using FlowJo software (FlowJo LLC, OR, USA).
